# Surgical reconstruction for fibular hemimelia

**DOI:** 10.1007/s11832-016-0790-0

**Published:** 2016-12-01

**Authors:** Dror Paley

**Affiliations:** Paley Institute, 901 45th St., West Palm Beach, FL 33407 USA

**Keywords:** Fibular hemimelia, SHORDT, Paley classification, Subtalar coalition, Fibular anlage, SUPERankle procedure

## Abstract

Fibular hemimelia presents with foot deformity and leg length discrepancy. Previous classifications have focused on the degree of fibular deficiency rather than the type of foot deformity. Published methods of surgical reconstruction have often failed due to residual or recurrent foot deformity. The purpose of this report is to introduce new classification and reconstruction methods. The Paley SHORDT procedure is used to stabilize the ankle when there is a hypoplastic distal fibula with a dynamic valgus deformity. It involves shortening and realignment of the distal tibia relative to the fibula. In contrast, the Paley SUPERankle procedure is used when there is a fixed equinovalgus foot deformity. The SUPERankle uses a supramalleolar shortening-realignment osteotomy and/or subtalar osteotomies with anlage resection. Due to the bony instead of soft tissue correction of deformity, residual or recurrent deformity is prevented. Weakening of gastro-soleus and peroneal muscles is avoided by shortening of the tibia instead of tendon lengthening. The limitation of ankle motion is related to ankle dysplasia rather than surgery or lengthening. A plantigrade-stable foot and ankle leads to an excellent functional result comparable or better than a Syme’s amputation with prosthetic fitting. Serial lengthening procedures combined with the SHORDT or SUPERankle reconstruction lead to limb length equalization with a plantigrade, painless, functional foot.

## Introduction

Fibular hemimelia (FH) is a congenital deficiency where part or all of the fibular bone is hypoplastic, dysplastic or aplastic associated with hypoplasia and dysplasia of the tibia and hypoplasia, dysplasia and aplasia of parts of the foot. The phenotype has a wide spectrum of pathology, ranging from mild to severe limb length discrepancy, ankle/foot deformities with or without subtalar coalition, midfoot coalitions and absent rays. Knee ligament deficiencies and knee valgus deformity as well as associated femoral hypoplasia, dysplasia and partial aplasia are common. It is therefore part of the same spectrum of deficiency as congenital femoral deficiency. These are commonly referred to as postaxial deficiencies and are distinct in their pattern from preaxial deficiencies such as tibial hemimelia.

FH occurs in between 1:135,000 and 1:50,000 births [1–3]. Bilateral FH (fibular hemimelia affecting both legs) occurs much less commonly. The etiology of FH remains unknown, and in most cases it is usually not an inheritable condition, with the vast majority of children born with this condition having no family history of other birth defects. The exception to this is when FH is associated with deficiency in more than one limb; for example, bilateral FH is often an autosomal dominant condition. When multiple limbs are affected by a limb deficiency, one can often assume that this was either an autosomal-dominant gene disorder (inherited or new mutation) or related to a teratologic agent (drug, radiation, virus, etc.). FH has been reproduced in a mouse model [4], suggesting that in most cases it may be a somatic gene mutation, although this theory has not been confirmed.

Children with FH have five main problems with their affected limb:

- limb length discrepancy
- foot and ankle deformities and deficiencies
- tibial deformity
- genu valgum
- knee instability

### Limb length discrepancy

 Unilateral FH leads to a limb length discrepancy due to inhibition of growth of the tibia and foot. In addition, many children with FH have some femoral growth inhibition (congenital femoral deficiency). The foot grows shorter in height, contributing to limb length discrepancy, but it is also shorter in length. This limb length discrepancy follows a Shapiro 1a curve, meaning its growth inhibition remains constant [5]. This characteristic makes the leg length discrepancy of FH predictable using the Anderson and Green [6], Moseley straight line graph [7], Amstutz method [8] or Paley Multiplier method [9]. The limb length discrepancy with FH ranges from very mild to very severe inhibition, ranging at maturity of the patient from 2 to 25 cm in the absence of femoral deficiency discrepancy. With combined inhibition of the femur and tibia the magnitude of leg length discrepancy at maturity can be >30 cm.

### Foot and ankle deformities

 Foot and ankle deformities have been the most challenging and disabling problems with FH. FH foot deformity has many components. At the ankle there is a dysplasia of the distal tibia and of the talus, which ranges from mild valgus of the distal tibia to severe dysplasia with flat malformed, maloriented joint surfaces. The distal tibial physis is more affected then the proximal tibial physis, with the former being often wedge shaped. The joint surface of the distal tibia ranges from a normal plafond with a 90° lateral distal tibial angle (LDTA) and 80° anterior distal tibial angle (ADTA) to a valgus plafond with an LDTA of <90° and an ADTA of >80° (procurvatum). The distal tibial articular surface is often concave in the frontal plane as part of a ball and socket ankle joint. The talus too ranges in its articular shape from normal to ball shaped in the frontal plane and from round to nearly flat in the sagittal plane. The talar neck may be very short and have little concave offset. The ankle joint function with FH may range from: normal range of motion, stable, no valgus instability, and no deformity; to, limited arc of motion, unstable with valgus instability, and fixed equino-valgus or varus deformity. Part of this deformity and instability is related to the fibular deficiency and part to the subtalar pathology. The fibula normally contributes to the lateral stability of the ankle. If the fibula is absent or deficient, then the ankle will sublux or roll into valgus. The subtalar joint pathology ranges from a normal subtalar joint to a subtalar joint with subtalar coalition. This subtalar coalition usually involves the posterior facet and is often malunited into equino-valgus. In a small minority of cases the subtalar coalition is malunited into equino-varus (clubfoot type). The combination of a malunited coalition, with valgus ankle joint instability, with a maloriented distal tibia produces a very significant magnitude of equino-valgus deformity of the foot and ankle. This foot malorientation is also associated with contractures of the tendo-Achilles and peroneal tendons. A further tether into equino-valgus may come from the fibular remnant referred to as the anlage. This anlage may be fibrous or both fibrous and cartilaginous. In some cases there is coalition of the cartilaginous fibular anlage to the calcaneus. Much of this patho-anatomy can be well visualized using magnetic resonance imaging (MRI).

Beyond the hindfoot deformities there can be deformities of the midfoot. When midfoot deformity is present it is most commonly abductus and rockerbottom. Most midfoot deformities are most commonly related to coalition between the cuboid and calcaneus. Talo-navicular joint coalition can also be present. One or more rays may be missing, making the foot narrower. Absence or weakness of the peroneus longus may lead to overpull of the tibialis anterior and elevation of the first metatarsal with compensatory flexion of the first metatarsophalangeal joint (dorsal bunion). A bracket first metatarsal or a bracket conjoined first and second metatarsal with hallux varus is not uncommon. Syndactaly between some or all of the toes is also common.

### Tibial deformity

 There is often a mild to severe diaphyseal tibial deformity of the valgus-procurvatum. A skin dimple is usually present over the apex of this angulation. The fibular anlage is located like the string of a bow in a straight line opposite the concavity of this deformity. This thick fibro-cartilagenous remnant may contribute to this angulation by tethering the growth of the tibia on its posterior-lateral side.

### Knee joint deformities

 The knee joint frequently has a valgus deformity. This valgus is related both to the distal femur and the proximal tibia. The lateral epiphysis of the proximal tibia may be delayed in its ossification compared to the normal opposite side.

### Knee instability

 Many patients with FH have hypoplasia or aplasia of the anterior and or posterior cruciate ligaments. The tibia may be subluxed anteriorly relative to the femur. The ligament deficiency and subluxation are often not symptomatic at a young age, but these become a bigger problem when the child becomes taller and heavier. Patients with anterior subluxation may have associated a rounded posterior aspect of the proximal tibial epiphysis. Whether this is primary (congenital) or secondary (developmental) is unclear.

## Classification of FH (Fig. 1)

Fibular hemimelia is not one condition where all of the cases have the same amount of deformity or deficiency or limb length discrepancy. Consequently, to facilitate the physicians’ recommendation of a specific treatment, FH is classified into different groups according to degree of severity. There are numerous classifications of FH [10–16], with the majority of these limited by the fact that they were developed at a time when surgical reconstruction for FH was unsuccessful and when amputation was the primary or only consideration for treatment. Therefore, the different groups of FH that have been described in the various classifications do not relate to the different types of treatment that are currently available. Most are only descriptive and recommend Syme’s amputation independent of the type of FH. The most commonly used classification is that of Achterman and Kalamchi [11], which describes the amount of fibular deficiency. We now know that the amount of leg length discrepancy and foot deformity, which are the two biggest problems in FH, do not correlate to the amount of fibula that is missing. The best prognostic factor is the foot deformity itself. Therefore, a classification based on the foot deficiency is needed. Birch et al. [13] classified FH according to the number of rays of the foot and recommended amputation for most cases with less than three rays.

**Fig. 1:**
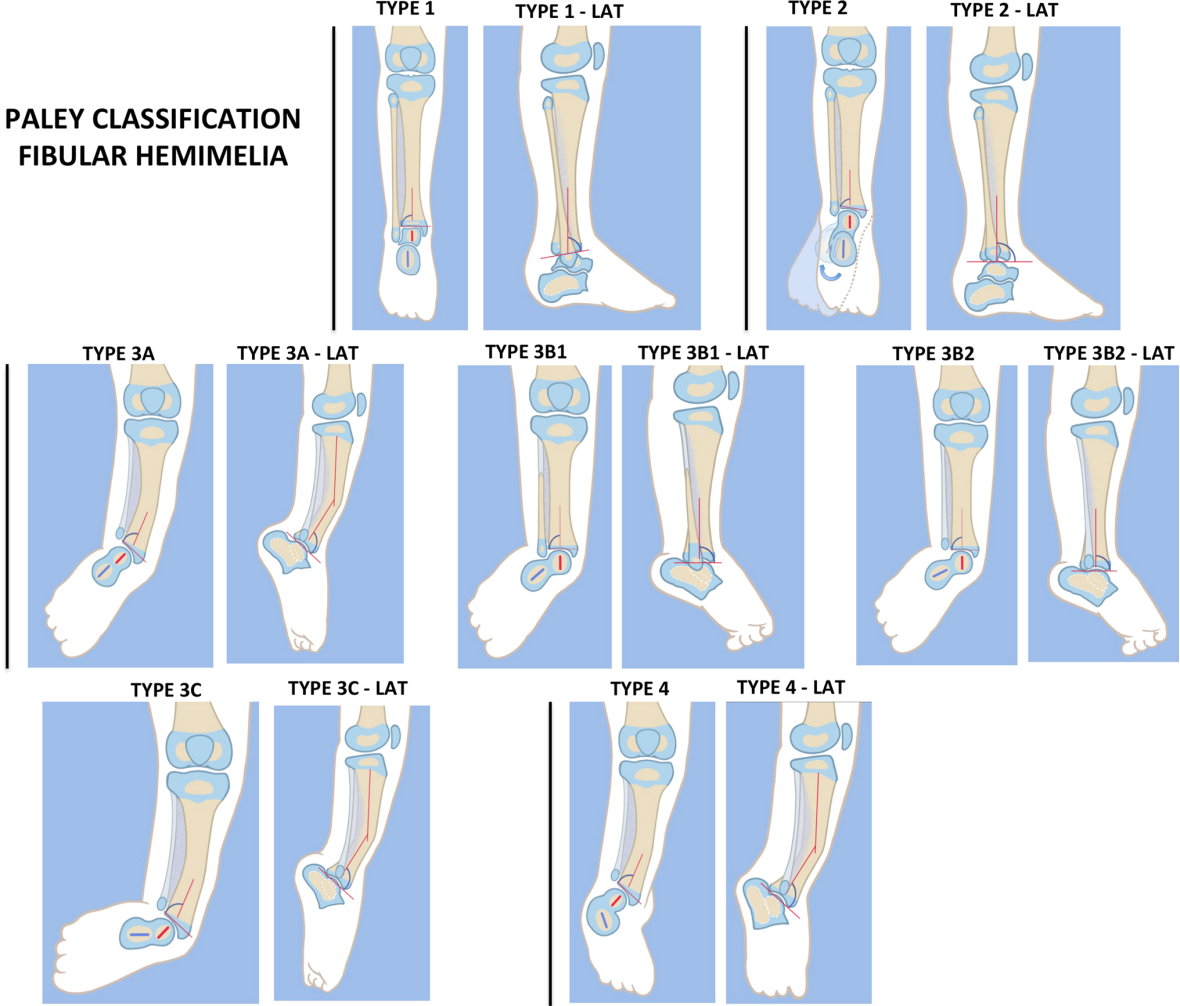
Paley classification of fibular hemimelia (FH).* Type 1* Stable ankle,* Type 2* dynamic valgus ankle,* Type 3* fixed equino-valgus ankle, *3A* Ankle type, *3B* subtalar type, *3C* combined ankle/subtalar, * Type 4* fixed equino-varus ankle. *LAT* lateral. Reproduced with permission by the Paley Foundation

The Paley classification (Fig. 1) [15, 16] is the first classification of FH to be designed with reconstructive surgery options in mind. It is based on the patho-anatomy and deformities of the ankle and subtalar joint. Each Paley classification type has a different surgical treatment; it is independent of the number of rays or the leg length discrepancy. The Paley classification of FH describes of four types of FH, with type 3 subdivided into three subtypes, as shown in the following list.

- Type 1: stable ankle. In many cases the ankle of type 1 cases appears completely normal, and the fibula is only slightly shorter at its upper end compared to the opposite side. There are some type 1 cases with complete fibular aplasia. The predicted leg length discrepancy in type 1 cases is typically less than 5 cm (2 in.).
- Type 2: dynamic valgus The foot in these cases can be brought into a plantigrade position. There is no fixed equino-valgus. Most feet have a ball and socket ankle joint with a fibula that is relatively short compared to the tibia at the level of the ankle joint. The normal fibula has its distal physis at the level of the ankle mortise. When the fibula is short distally, its distal physis is proximal to the ankle joint. While the foot can be placed plantigrade, the ankle naturally rolls outwards, and the patient stands and walks in valgus. There is often limited dorsiflexion in this group but not fixed equinus.
- Type 3: fixed equino-valgus There is a fixed deformity of equino-valgus. In some cases the foot can be brought out of equinus with obligatory valgus. When the heel is held out of valgus in a neutral position, there is a fixed equinus deformity. This fixed equino-valgus can be divided into three groups:

- Type 3A: ankle type. The fixed equino-valgus deformity is due to a malorientation of the ankle joint (distal tibial epiphysis is in procurvatum-valgus; the LDTA is decreased and ADTA is increased).
- Type 3B: subtalar type. There is a malunited subtalar coalition. The calcaneus is located lateral to the talus and is often tilted into valgus relative to the body of the talus. If there is a fibula with distal fibular physis and lateral malleolus present (3B1), it is proximally migrated and articulates with the dorsal surface of the calcaneus. The same deformity can occur without a fibula (3B2).
- Type 3c: combined subtalar and ankle type. Both distal tibial malorientation and malunited subtalar coalition are present.

- Type 4: fixed equino-varus The only difference between type 3B or 3C and type 4 is that the subtalar coalition is malunited in varus in the former. In most of these cases the distal tibia is also maloriented into procurvatum and valgus. This type can be misdiagnosed as a clubfoot. It its resistant to Ponsetti casting as well as clubfoot releases of the subtalar joint since there is a subtalar coalition.

## Reconstructive life plan

The surgical treatment of FH is designed to address all of the deformities and deficiencies and length discrepancies. The first step in this process is to create a reconstructive life plan individualized for each patient. This involves evaluating all of the surgical deformities and deficiencies, predicting the limb length discrepancy at maturity and then coming up with a surgical plan to correct these in the fewest number of surgeries spread out as much as possible throughout the child’s growing years, so that by skeletal maturity the child has achieved equal leg length, a functional plantigrade foot, excellent alignment of the hip, knee and ankle and, as needed, a stable knee joint.

### Step 1: predicting leg length discrepancy and determining the number of lengthening surgeries

The first step is measuring the leg length discrepancy using standing radiographs of both lower limbs, with the short leg on a lift of known amount [17]. The total leg length discrepancy at skeletal maturity and the separate bone segment (femur, tibia, foot height) discrepancy at maturity can be calculated using the multiplier method for limb length discrepancy prediction [18]. The multiplier method has been validated for accuracy in the prediction of congenital limb length discrepancy, including for FH [19, 20]. It is now possible to do this method using smart phone apps [App name 1: Paley Growth (OS1 only); App name 2: Multiplier (OS1 and Android)]. Once the predicted leg length discrepancy at skeletal maturity has been calculated, a determination of the number of limb length equalization procedures can be made.

Under the age of 4 years it is safe to lengthen up to 5.0 cm in the tibia; lengthening of >5.0 cm can lead to growth inhibition in young children [21]. Subsequent lengthenings can be performed preferably 4 years apart as needed to achieve limb length equalization at skeletal maturity. Lengthenings performed at an older age can safely achieve up to 8.0 cm of lengthening. Therefore, one lengthening by age 4 years and one at age 8 years would achieve a total lengthening of 13 cm (5.1 in.) (5.0 + 8.0 cm). One lengthening by age 4 years plus one at age 8 years and one at age 12 years would achieve a total lengthening of 21.0 cm (8.25 in.) (5 + 8 + 8 cm). If additional equalization is required, epiphysiodesis of the opposite proximal tibia can always be considered. Epiphysiodesis is typically performed at a specific age calculated with the Paley multiplier formulae and is usually recommended for up to 5.0 cm (2 in.) of limb length equalization. Therefore, leg length equalization up to 26.0 cm can be achieved with three lengthenings (21 cm) plus an epiphysiodesis (5 cm). This treatment covers the majority of cases with limb length discrepancy due to FH. It is rarely ever necessary to perform more than three limb lengthening procedures to equalize limb length discrepancy due to FH. Cases that present with discrepancies of >25.0 cm usually have some shortening in the femur, which can be treated with simultaneous or independent lengthening of the femur. This treatment will be discussed in a later section.

### Step 2: determining the Paley type of FH

The next step is to determine what type of FH. This distinction is based on the clinical exam of the foot and ankle. If there is a fixed equino-valgus foot deformity, then it is a type 3. If there is a fixed equino-varus foot deformity, then it is a type 4. If the ankle deformity is dynamic, then it is a type 2. If there is no foot deformity and the ankle is stable, then it is a type 1. An MRI is not necessary to separate types 1, 2, 3 and 4; these types can be determined by clinical and radiographic examination. An MRI examination is helpful to subdivide the type 3 FH into subtypes a, b or c.

### Step 3: determining the surgical procedures required

 Most patients with type 1 FH do not require any foot surgery; rather, treatment consists of lengthening the tibia and fibula with no foot fixation. Most patients with type 2 will require a shortening realignment osteotomy of the distal tibia to correct the valgus and stabilize the ankle. This procedure is called the SHORDT (‘shortening osteotomy realignment distal tibia’). After the SHORDT, or together with it, the tibia can be lengthened. Types 3 and 4 FH have fixed deformities that should be corrected early to allow the patient to walk with the foot in a plantigrade position and to be able to wear a shoe properly. It is important to correct this deformity either before or at the time of tibial lengthening. Types 3 and 4 are treated by the SUPERankle procedures (SUPER being an acronym for ‘systematic utilitarian procedure for extremity reconstruction’). The SUPERankle procedure was developed by the author in 1996. It is the most successful method to correct the fixed equino-valgus of type 3 FH or fixed equinovarus of type 4 FH. The SUPERankle procedure is performed in children between 18 and 24 months of age. It involves supramalleolar and/or subtalar osteotomies combined with soft tissue release. I have performed the SUPERankle in infants as young as 12 months and in adults as old as 32 years. Lengthening is often combined with the SUPERankle procedure.

### Example of reconstructive life plan

A 6-month-old boy presents with Paley type 3c FH. The predicted leg length discrepancy at skeletal maturity is 25.0 cm, with a valgus knee deformity. The reconstructive life plan would consist of:

- Surgery #1, at age 18 months, SUPERankle procedure combined with lengthening of 5.0 cm combined with hemiepiphysiodesis of distal femur for valgus knee correction.
- Surgery #2, at age 8 years, lengthening 7.0 cm of tibia.
- Surgery #3, at age 12 years, lengthening 8.0 cm of tibia.
- Surgery #4, at age 13 years, epiphysiodesis of the proximal tibia on long leg for correction of 5.0 cm.
- Total leg length equalization = 25 cm (10 in.).

By the end of the first consultation, the child’s parents have a roadmap for the future. This allows them to plan their lives around the surgical plan. They leave the first consultation with a good understanding of what it would take to successfully correct the foot and leg deformities and to equalize the limb length discrepancy by skeletal maturity. They can now make an educated decision whether to reconstruct and lengthen their child’s leg with FH.

## Biomechanical principles related to FH reconstructive surgery

The normal ground reaction force vector passes lateral to the center of the tibial plafond and talus because the point of contact of the calcaneus with the ground is lateral to the center of the ankle joint [22], resulting in a valgus moment arm on the ankle joint. This moment arm is normally resisted by the posterior tibial tendon during the stance phase of gait. The lateral moment arm is also blocked by the buttressing effect of the lateral malleolus. This interaction is the reason why even small amounts of loss of length or position of the fibula after ankle fracture can lead to lateral subluxation of the talus in the mortise and eventually ankle arthritis [23]. Elimination of the lateral shift of the talus in the mortis when the fibula is missing requires medialization of the ground reaction force vector by shifting the point of contact of the calcaneus with the ground. The buttress of the fibula is replaced by the varusized tibial plafond, since the lateral plafond is now more distal than the medial plafond. The talus wants to shift medially and loads the medial malleolus. If a hypoplastic fibula remnant is present it can be shifted distally or the tibia shortened relative to the fibula in order to relatively lengthen the fibula to the tibia and restore the buttress effect of the lateral malleolus. These biomechanical principles are the basis of the SUPERankle and SHORDT procedures [24].

## Methods

### SHORDT: SHortening Osteotomy Realignment Distal Tibia

The SHORDT (Figs. 2, 3, 4, 5, 6, 7, 8, 9, 10, 11, 12, 13, 14, 15, 16, 17) is a procedure that was designed by the author in 2014 to treat valgus instability of the ankle in patients who have a hypoplastic fibula where the growth plate of the distal fibula is present (Figs. 2 and 17). Although in theory it could also be used for a fibular remnant lacking a distal physis, such remnants are so hypoplastic and have little growth potential that they are not likely to remain a successful lateral buttress.

#### Surgical procedure

Step 1: Under tourniquet control make a medial longitudinal incision at the postero-medial border of the distal tibia extending past the ankle joint (Fig. 3).
Step 2: Incise the fascia covering the neurovascular bundle and decompress the posterior tibial nerve by cutting this fascia including the lacinate ligament [25] (Fig. 4).
Step 3: Expose the medial aspect of the tibia for approximately 5 cm up to the level of the distal physis (Fig. 5).
Step 4: Make a T-shaped incision in the periosteum and expose the bone. (Fig. 5).
Step 5: Make a second small longitudinal incision over the distal tibio-fibular syndesmosis. (Fig. 6).
Step 6: Cut the anterior tibiofibular syndesmotic ligament (Fig. 7a).
Step 7: Use a freer elevator to perforate and tear the posterior tibiofibular syndesmotic ligament (Fig. 7b).
Step 8: Use the elevator or scissors to release part of the distal interosseous membrane between the tibia and fibula. The tibia and fibula should separate apart after these releases (Fig. 8).
Step 9: Insert a guide wire in the frontal plane parallel to the valgus angle of the foot when it is dorsiflexed (parallel to plantar aspect of the foot) (Fig. 9).
Step 10: Insert another guide wire in the sagittal plane from anterior to posterior at a 10° tilt to the ankle joint to create an ADTA of 80° (Fig. 10). These wires define the plane of the distal osteotomy (Fig. 11).
Step 11: Measure the distance between the distal fibular physis and the ankle joint. This distance is the distance to be shortened. Insert a wire from the medial side perpendicular to the tibia at the shortening distance away from the distal tibial frontal plane wire at the lateral cortex. Add a second wire perpendicular to the tibia in the sagittal plane. Use these two wires to guide the proximal osteotomy plane of cut (Figs. 9 and 10).
Step 12: Make the first osteotomy along the proximal frontal plane wire perpendicular to the proximal tibial diaphysis (Figs. 9 and 10).
Step 13: Using a thin saw blade make the distal osteotomy parallel to the distal two wires along the frontal plane wire as its cutting surface (Fig. 11).
Step 14: Remove the trapezoidal segment of bone from the tibia (Figs. 12).
Step 15: Shorten the tibia relative to the fibula and temporarily fix it with two k-wires. The distal fibular physis should now be at the level of the ankle joint (Fig. 13).
Step 16: Fix the tibia with a small medial tibial locking T plate (Fig. 14).
Step 17: If the distal tibiofibular joint is not stable, insert a syndesmotic double washer compression suture such as a TightRope (Arthrex Inc., Naples, FL) or Ziptite (Zimmer Biomet Inc., Warsaw, IN) (Figs. 15, 16).

**Fig. 2:**
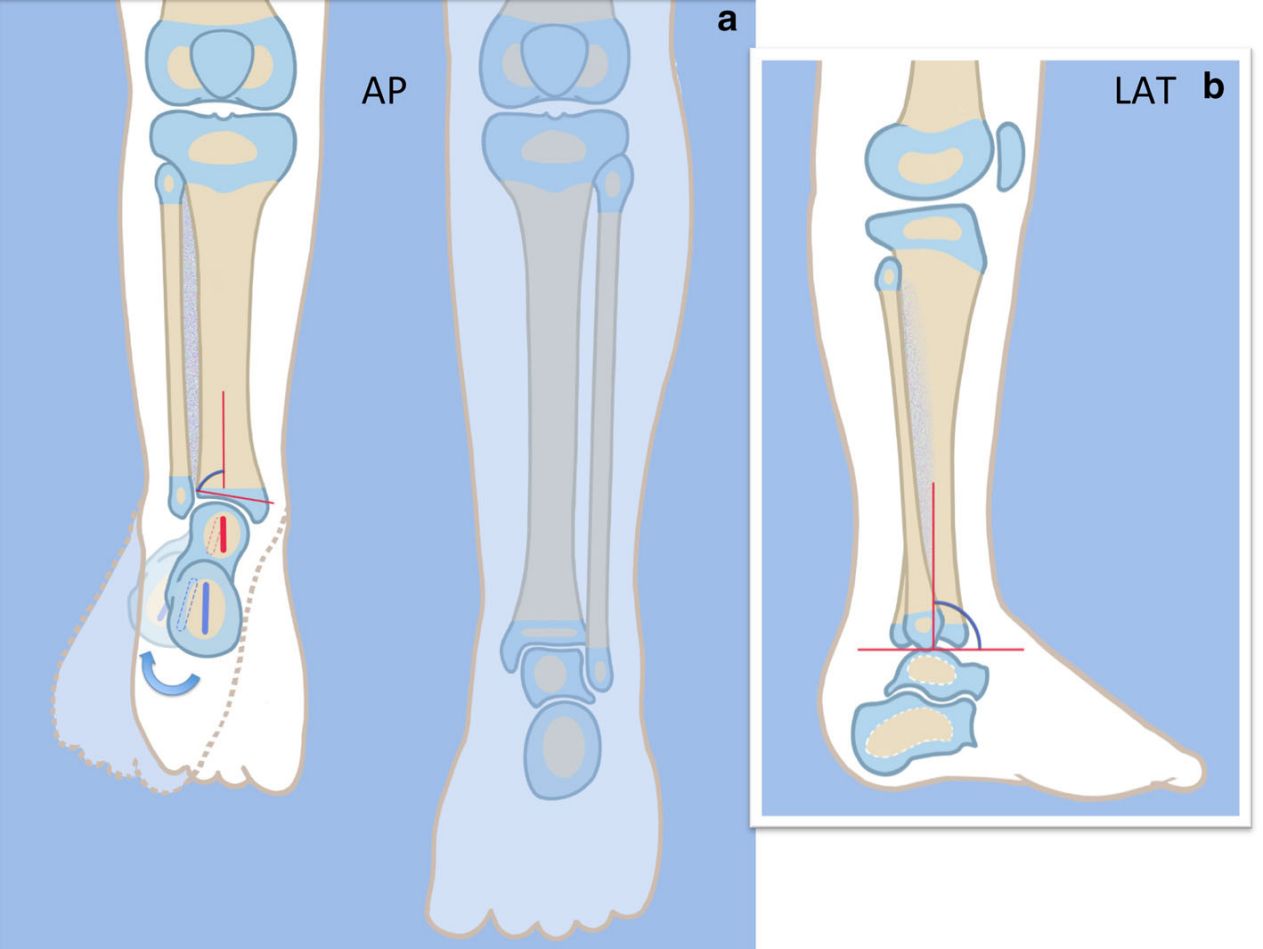
Paley type 2 FH. Dynamic valgus. The fibula is hypoplastic. The distal fibular physis is proximal to the level of the ankle joint. **a** Anteroposterior (*AP*) view illustration, **b** lateral (*LAT*) view illustration. The ankle joint is a ball and socket ankle. The ankle joint orientation is in valgus and procurvatum. The lateral distal tibial angle (LDTA) is <85° and the anterior distal tibial angle (ADTA) is >90°. Reproduced with permission by the Paley Foundation

**Fig. 3:**
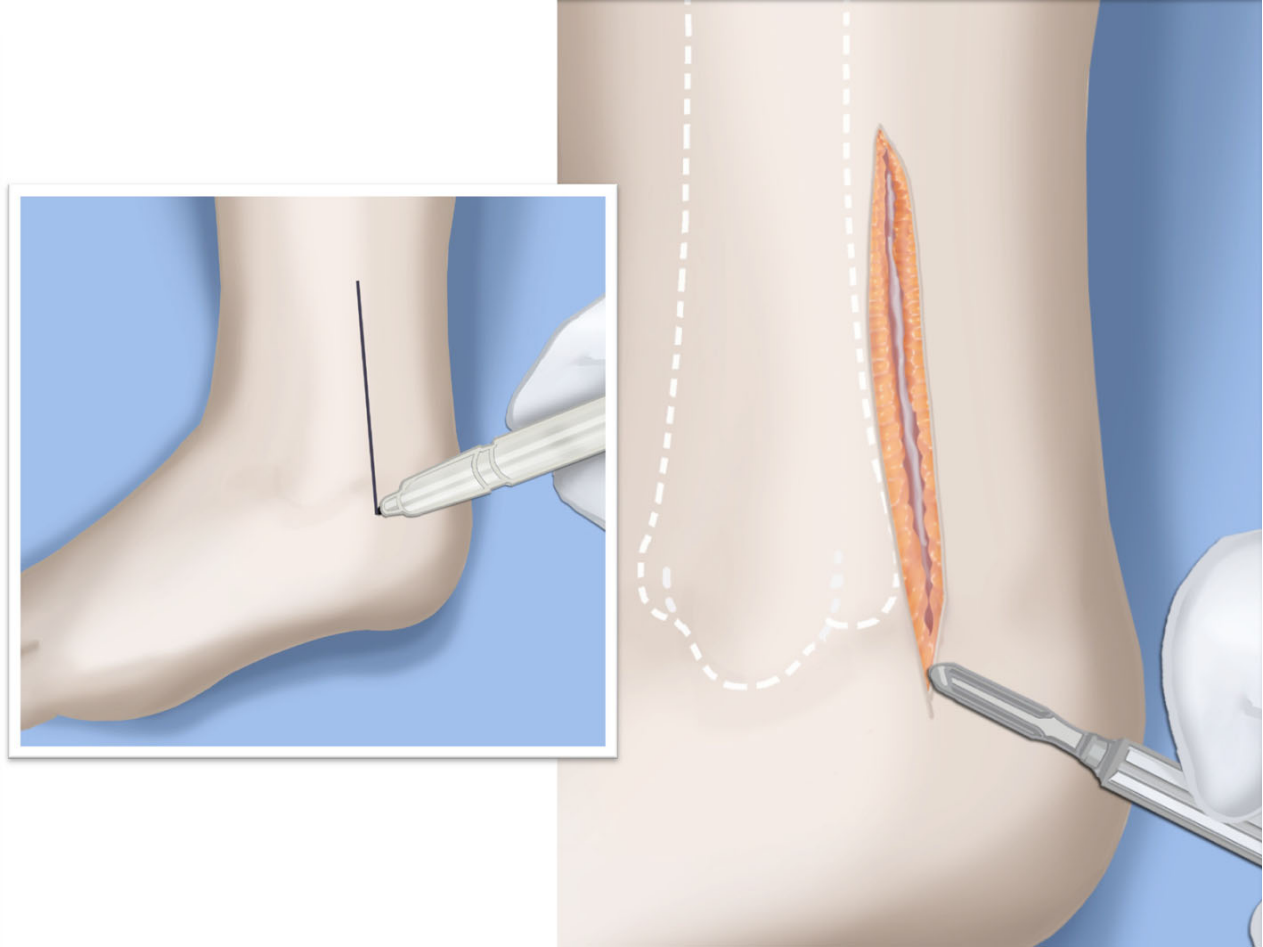
The incision for the SHORDT (‘shortening osteotomy realignment distal tibia’) is along the postero-lateral border of the tibia on the medial side. Reproduced with permission by the Paley Foundation

**Fig. 4:**
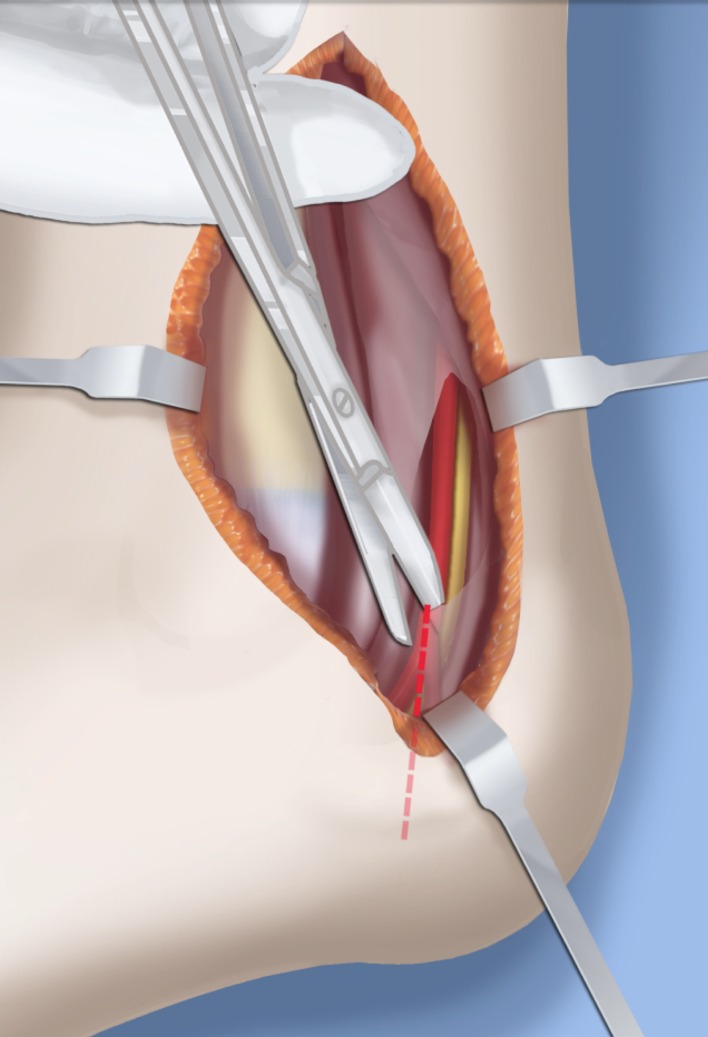
The tarsal tunnel is decompressed. Reproduced with permission by the Paley Foundation

**Fig. 5:**
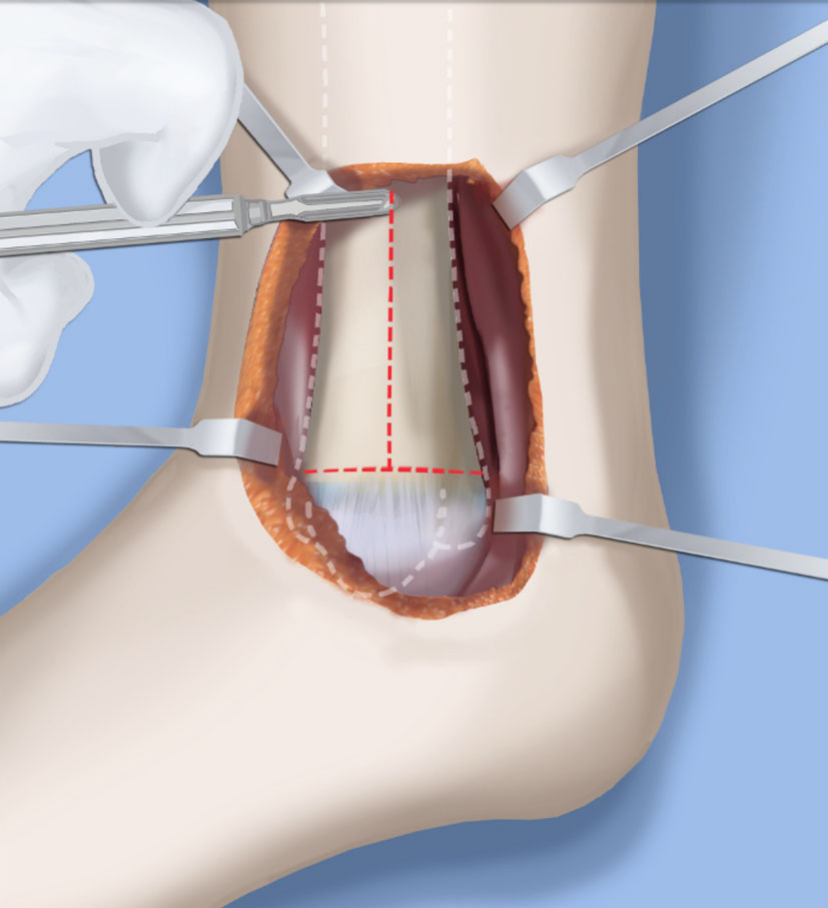
The periosteum of the tibia is cut in a T-shaped fashion, exposing the medial aspect of the distal tibia. Reproduced with permission by the Paley Foundation

**Fig. 6:**
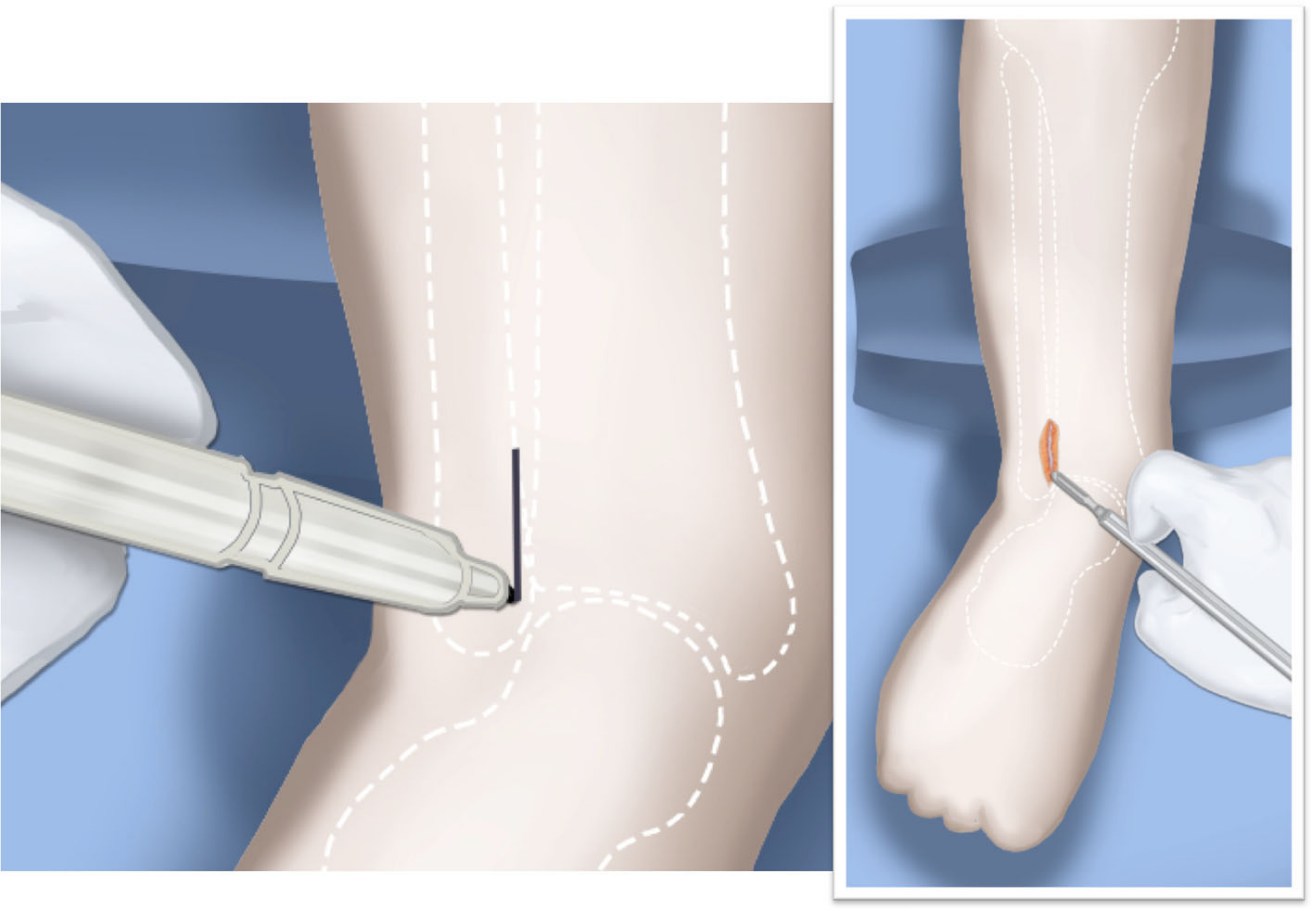
A second incision is made antero-laterally overtop the distal tibio-fibular syndesmosis. Reproduced with permission by the Paley Foundation

**Fig. 7:**
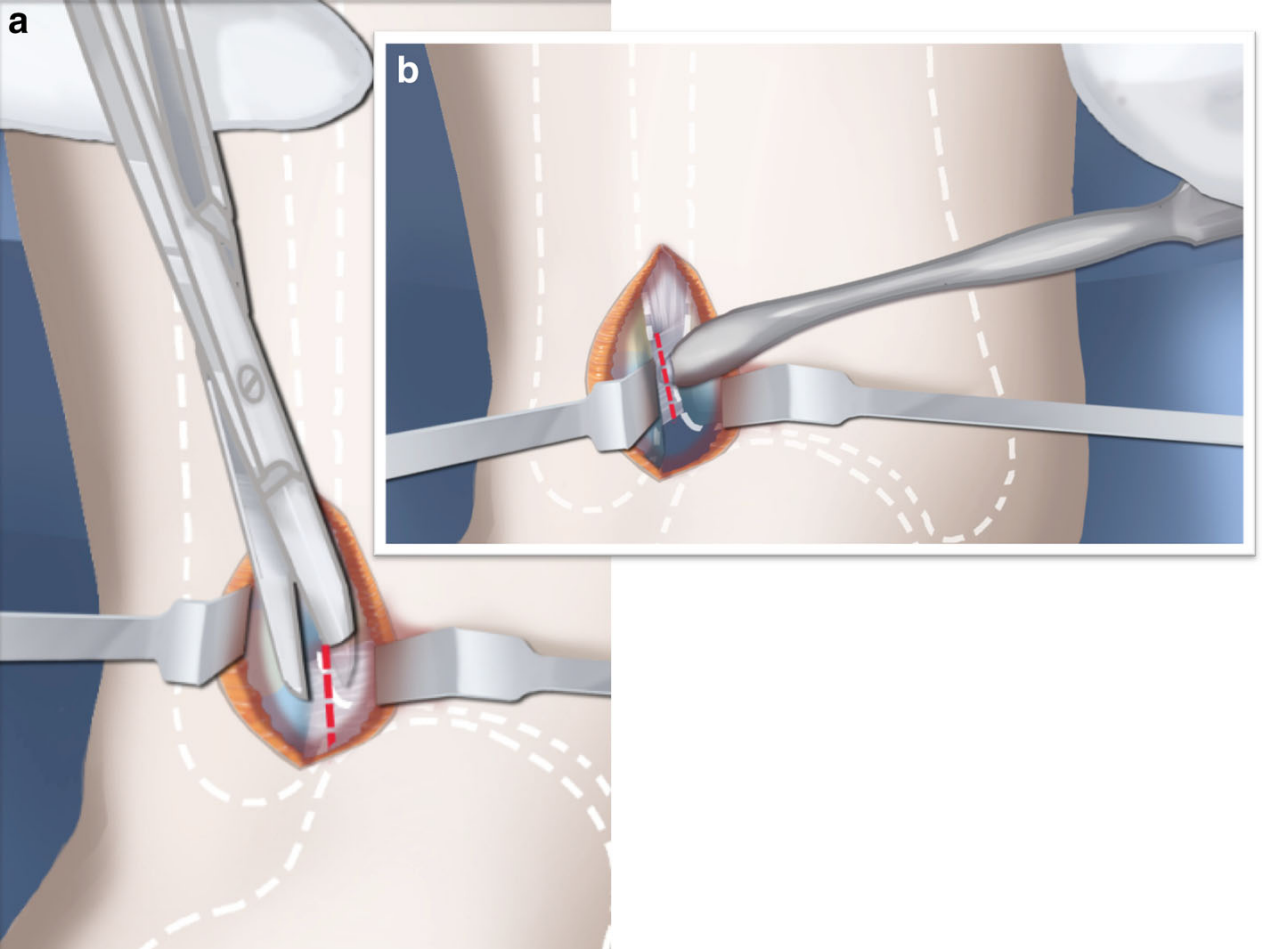
The anterior (**a**) and posterior (**b**) distal tibio-fibular syndesmostic ligaments are cut. Reproduced with permission by the Paley Foundation

**Fig. 8:**
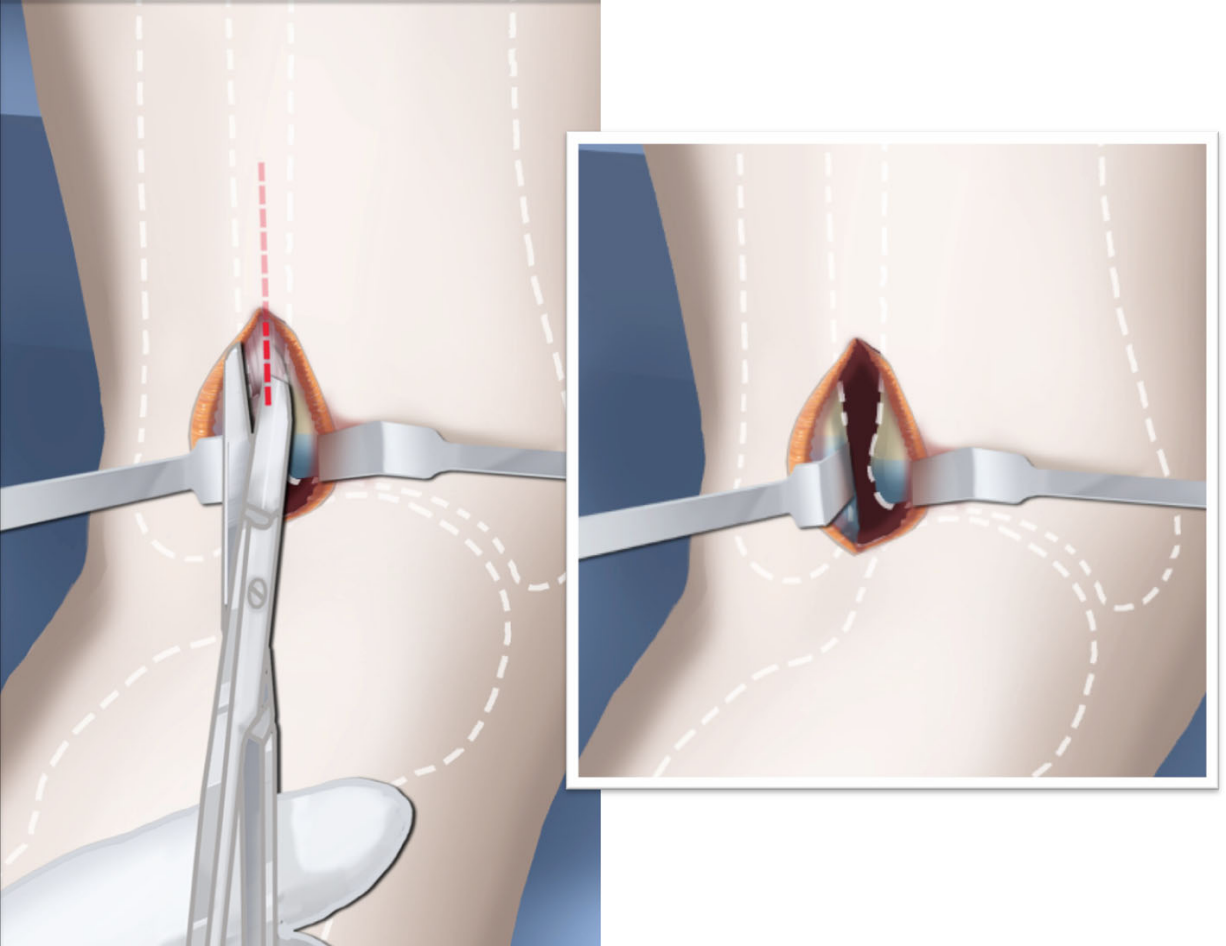
The interosseous membrane is also released through this incision. Reproduced with permission by the Paley Foundation

**Fig. 9:**
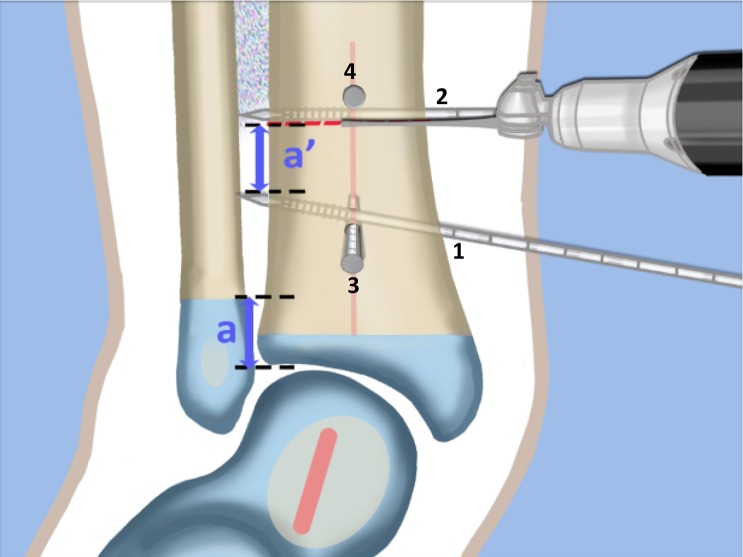
AP view. Insert one frontal plane (*1*, *2*) and one sagittal plane (*3*, *4*) guide wire at each level of planned osteotomy. These guide wires are inserted at the angle of the plane of the osteotomy. Distally, the frontal plane guide wire is parallel to the plantar aspect of the foot in its valgus position (*1*). Proximally, the frontal plane guide wire is perpendicular to the tibia (*2*). The distance between the two frontal plane wires at the lateral cortex (*a’*) is equal to the amount of planned shortening. This is based on the distance of the distal fibular physis from the joint line (*a*). Make the proximal osteotomy along the proximal frontal plane wire. Reproduced with permission by the Paley Foundation

**Fig. 10:**
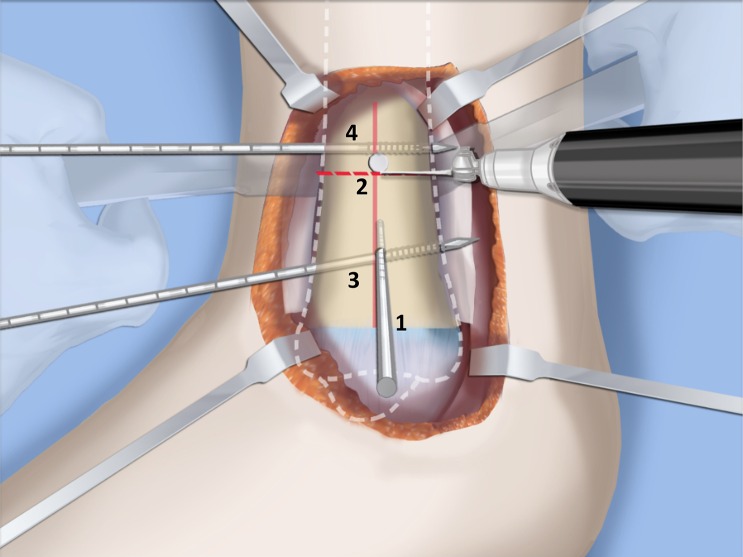
Medial View. The ADTA in the sagittal plane is 90° (plantar flexed) instead of the normal 80° (Fig. 2b). To correct this equinus deformity the distal sagittal plane guide wire (*3*) is oriented 10° plantar flexed to the joint line to simulate an ADTA 80°. Proximally the sagittal plane guide wire is perpendicular to the tibia (*4*). The proximal osteotomy is parallel to the proximal sagittal plane guide wire (*4*). Reproduced with permission by the Paley Foundation

**Fig. 11:**
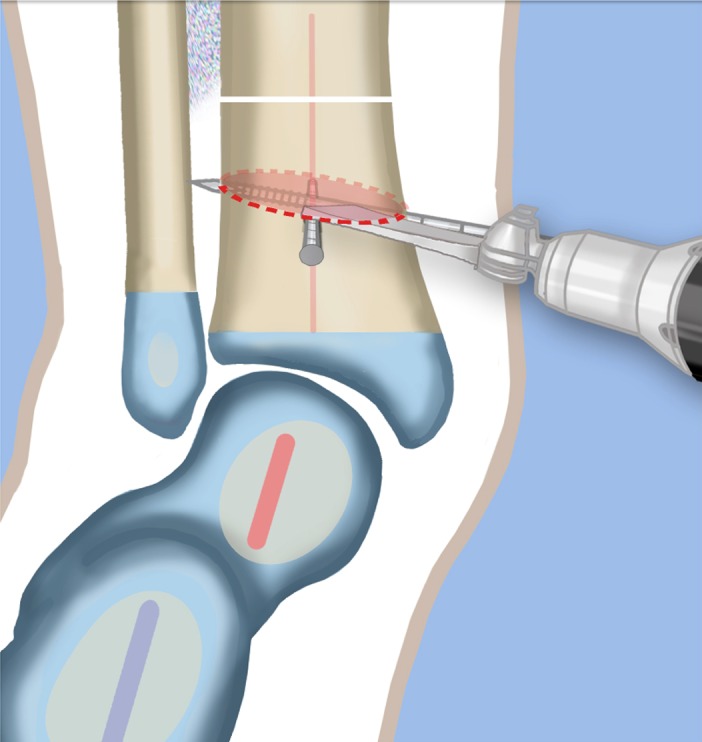
Make the distal osteotomy in the plane of the distal guide wires. Reproduced with permission by the Paley Foundation

**Fig. 12:**
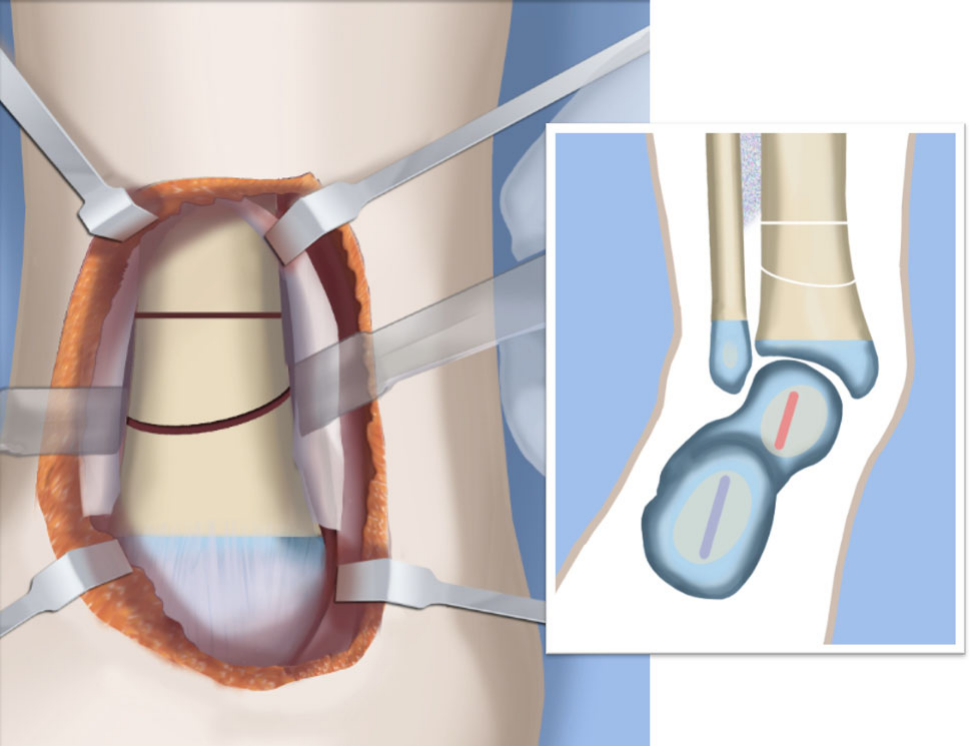
Both osteotomies are completed creating a trapezoidal segment of bone. Reproduced with permission by the Paley Foundation

**Fig. 13:**
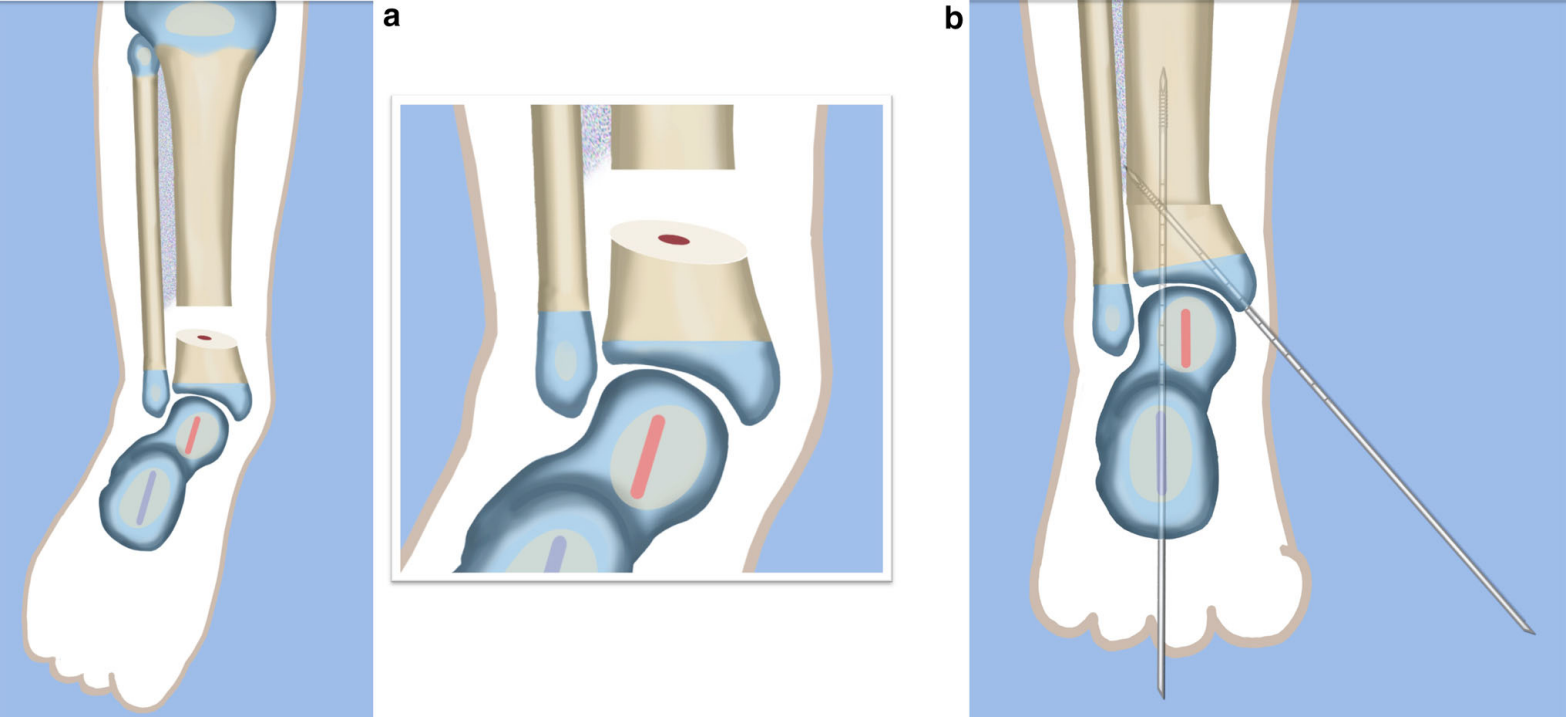
The trapezoidal segment of bone is removed (**a**) and the tibia is shortened and realigned relative to the fibula (**b**). Temporary k-wire fixation is used to hold it in place. Reproduced with permission by the Paley Foundation

**Fig. 14:**
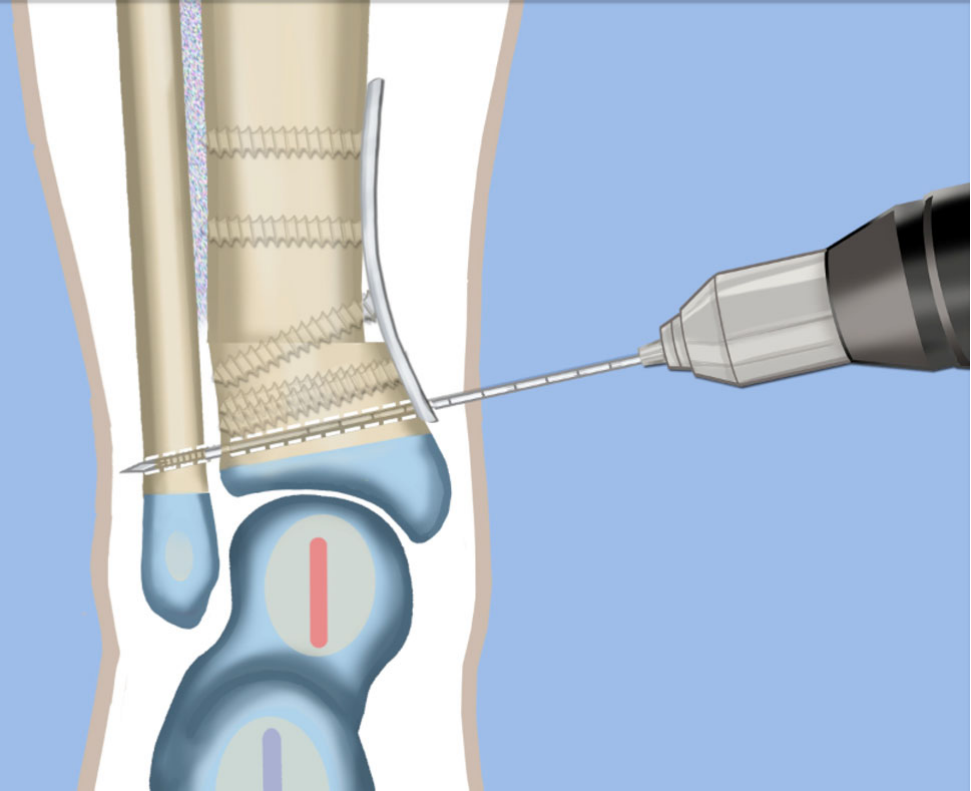
The distal tibia is plated from the medial side with a low profile locking plate. The distal tibia and fibula are drilled in order to pass a syndesmotic suture. Reproduced with permission by the Paley Foundation

**Fig. 15:**
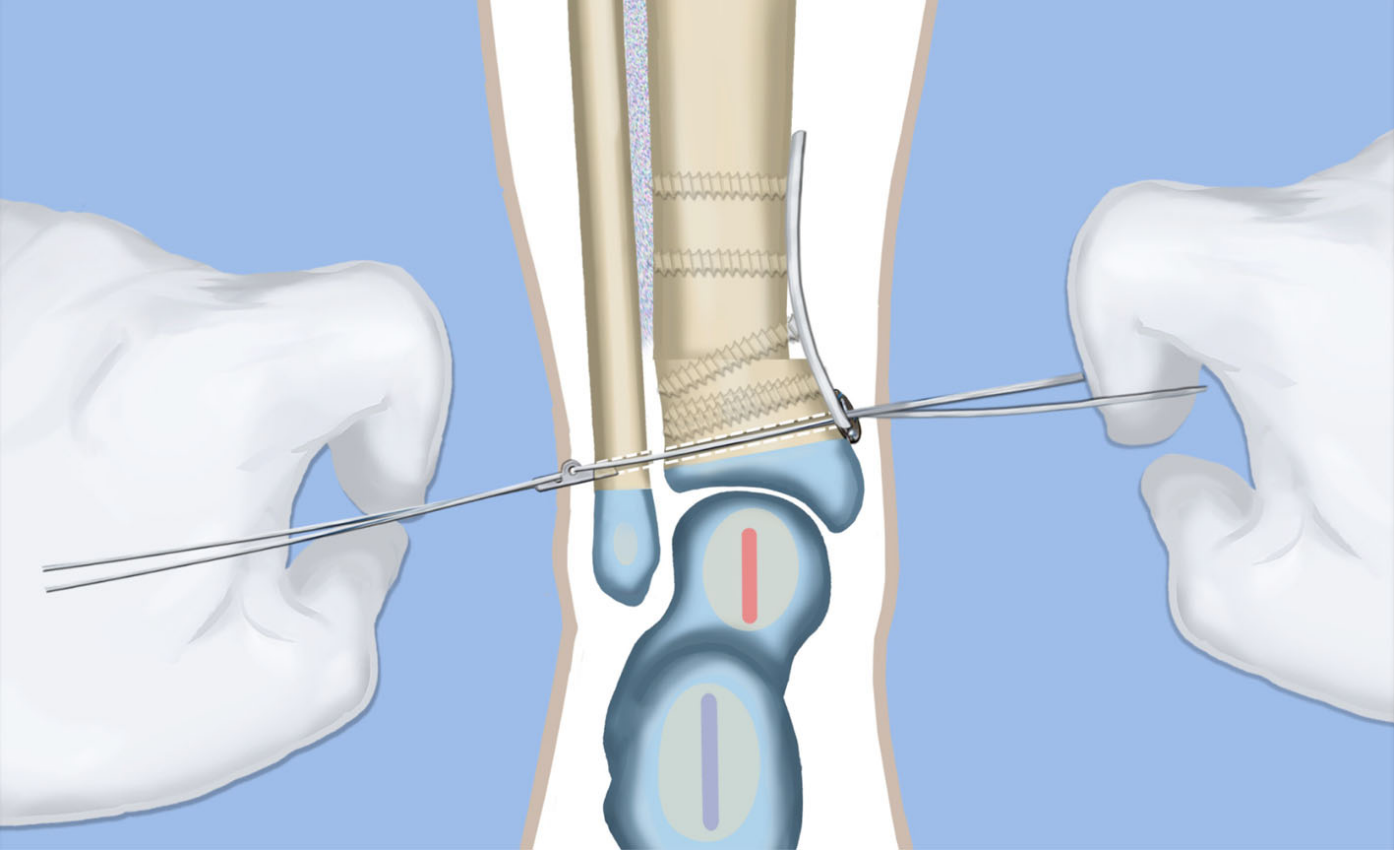
The syndesmotic suture is passed and the tibia is compressed to the fibula. Reproduced with permission by the Paley Foundation

**Fig. 16:**
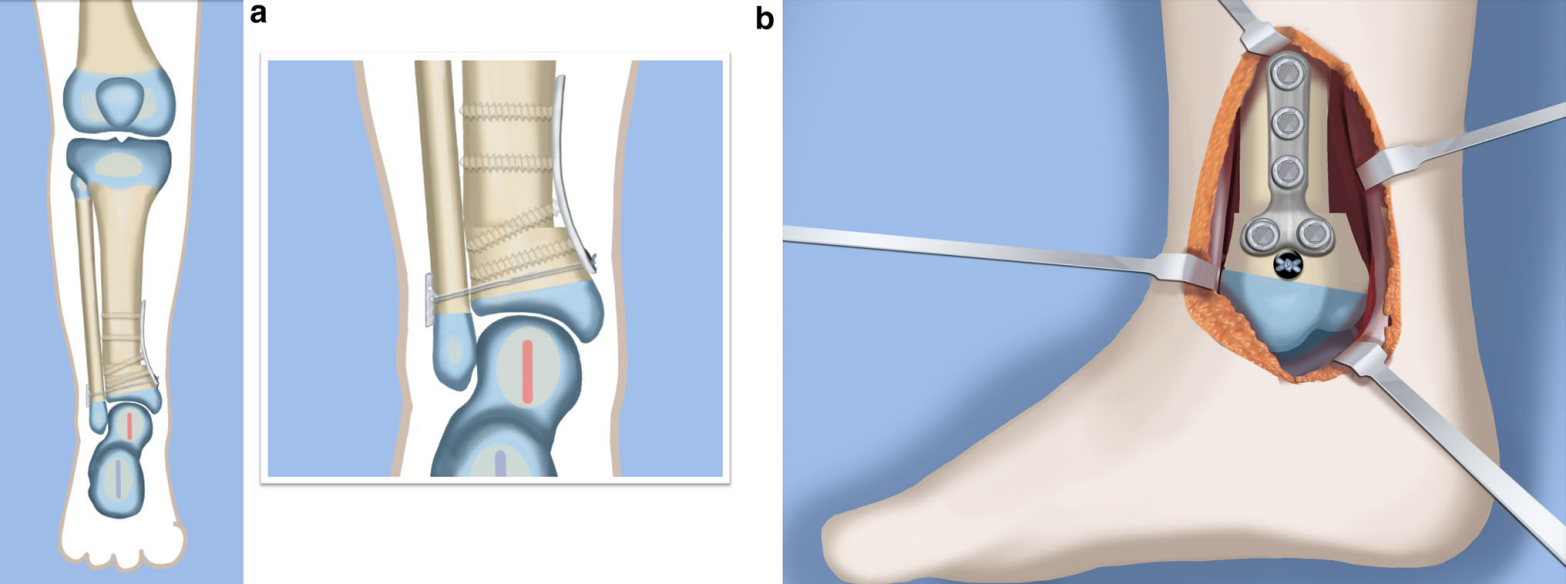
The distal tibial and fibular fixation is shown from anterior (**a**) and from the medial (**b**) views. The foot is fully realigned and stable. Reproduced with permission by the Paley Foundation

**Fig. 17:**
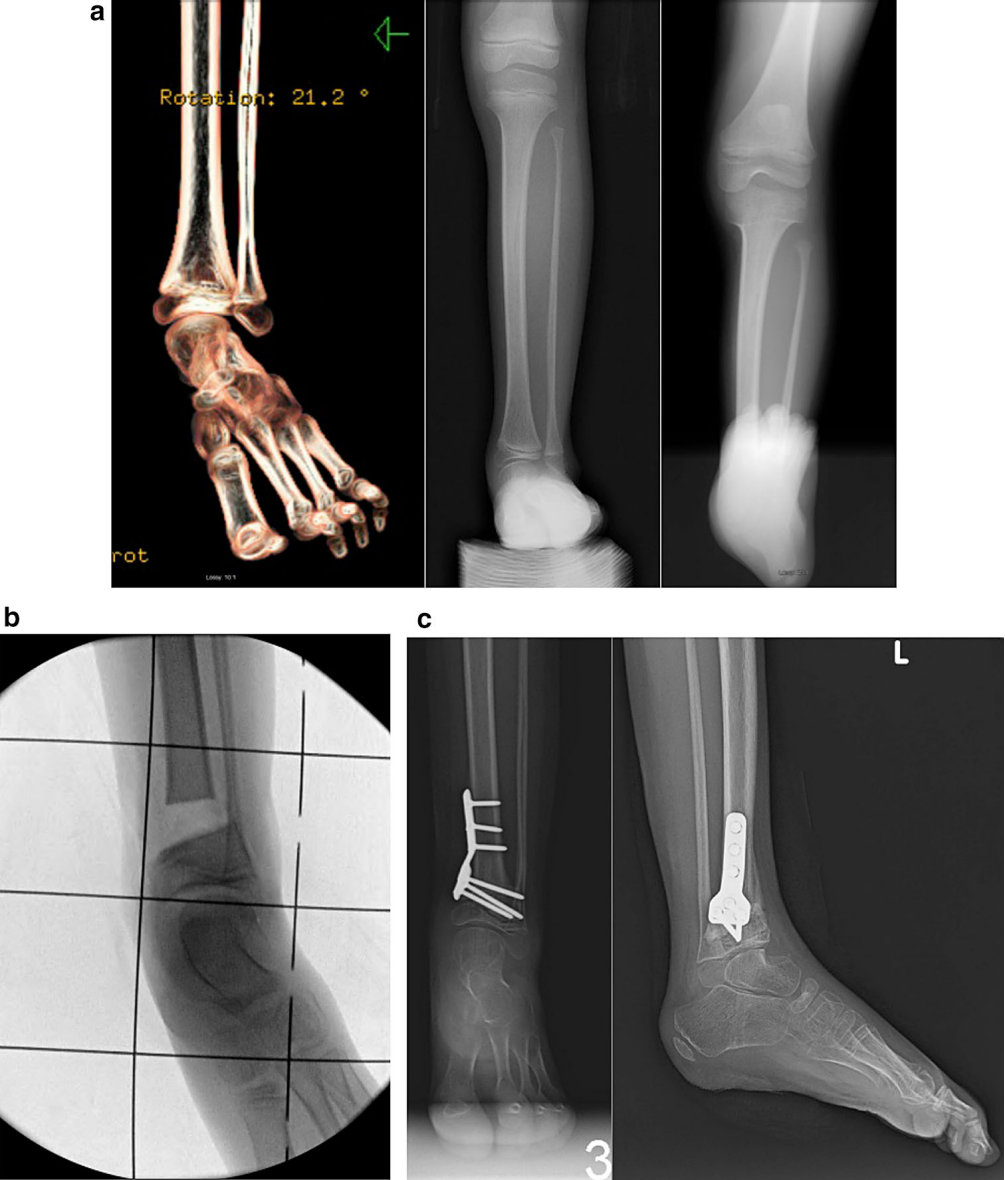
**a** Radiographs (*right* and *center*) and three-dimensional computed tomography image (*left*) of ball and socket ankle joint in patient with Paley type 2 FH. The heel is in valgus. The distal fibular physis is proximal to the ankle joint. **b** Intraoperative radiograph showing trapezoidal resection of distal tibia. **c** AP and lateral radiographs after acute shortening and internal fixation with locking plate. The distal tibial physis is at the ankle joint level

### SUPERankle procedure: Systematic Utilitarian Procedure for Extremity Reconstruction

**Fig. 18:**
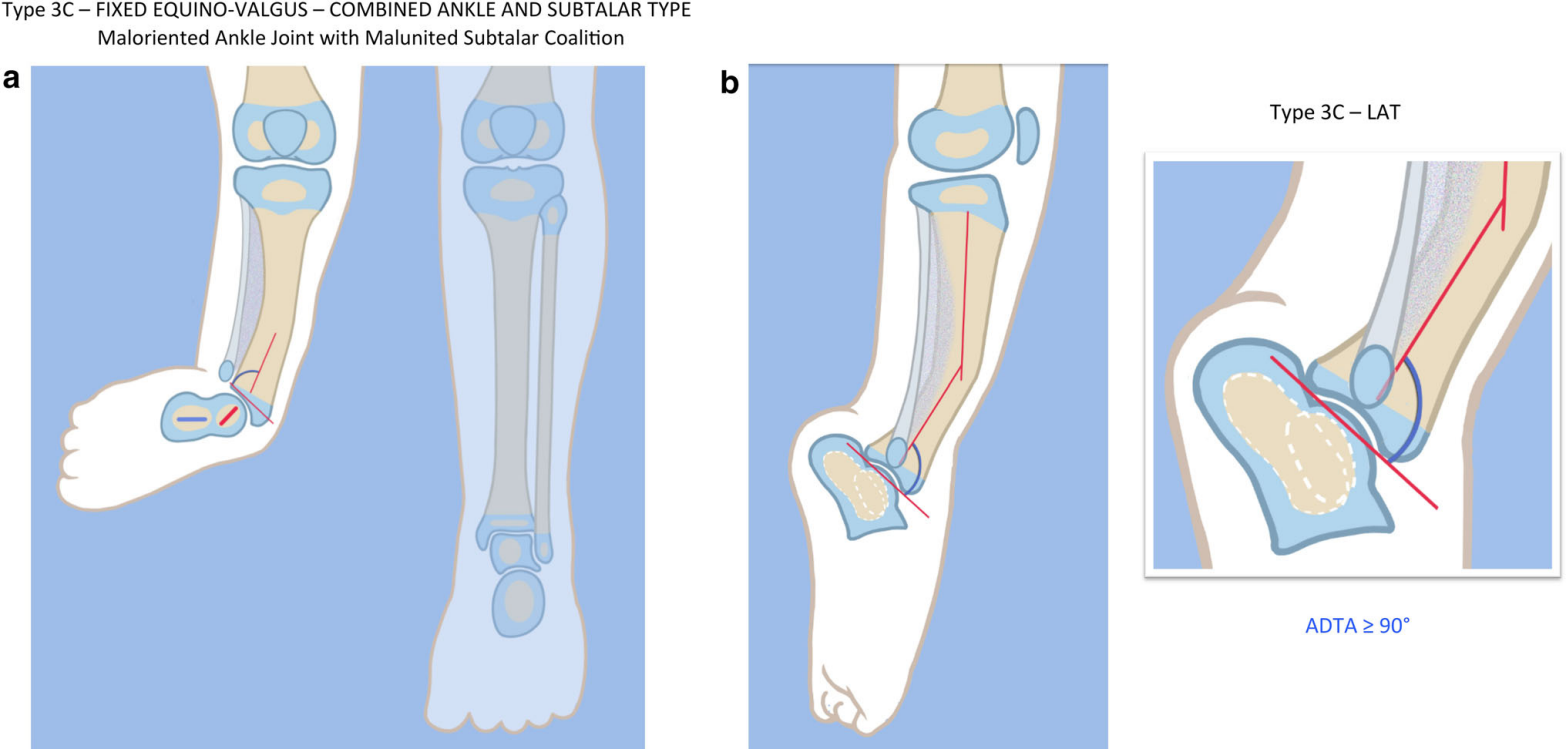
**a**, **b** Paley type 3c fibular hemimelia. The foot is in fixed equino-valgus. There is a fibrous and cartilaginous fibular anlage. There is a malunited subtalar coalition. The calcaneus is laterally translated and valgus (**a**). The tibial plafond is maloriented into valgus procurvatum (**b**). The tibial diaphysis has an antero-lateral bow. There is a leg length discrepancy. Reproduced with permission by the Paley Foundation

**Fig. 19:**
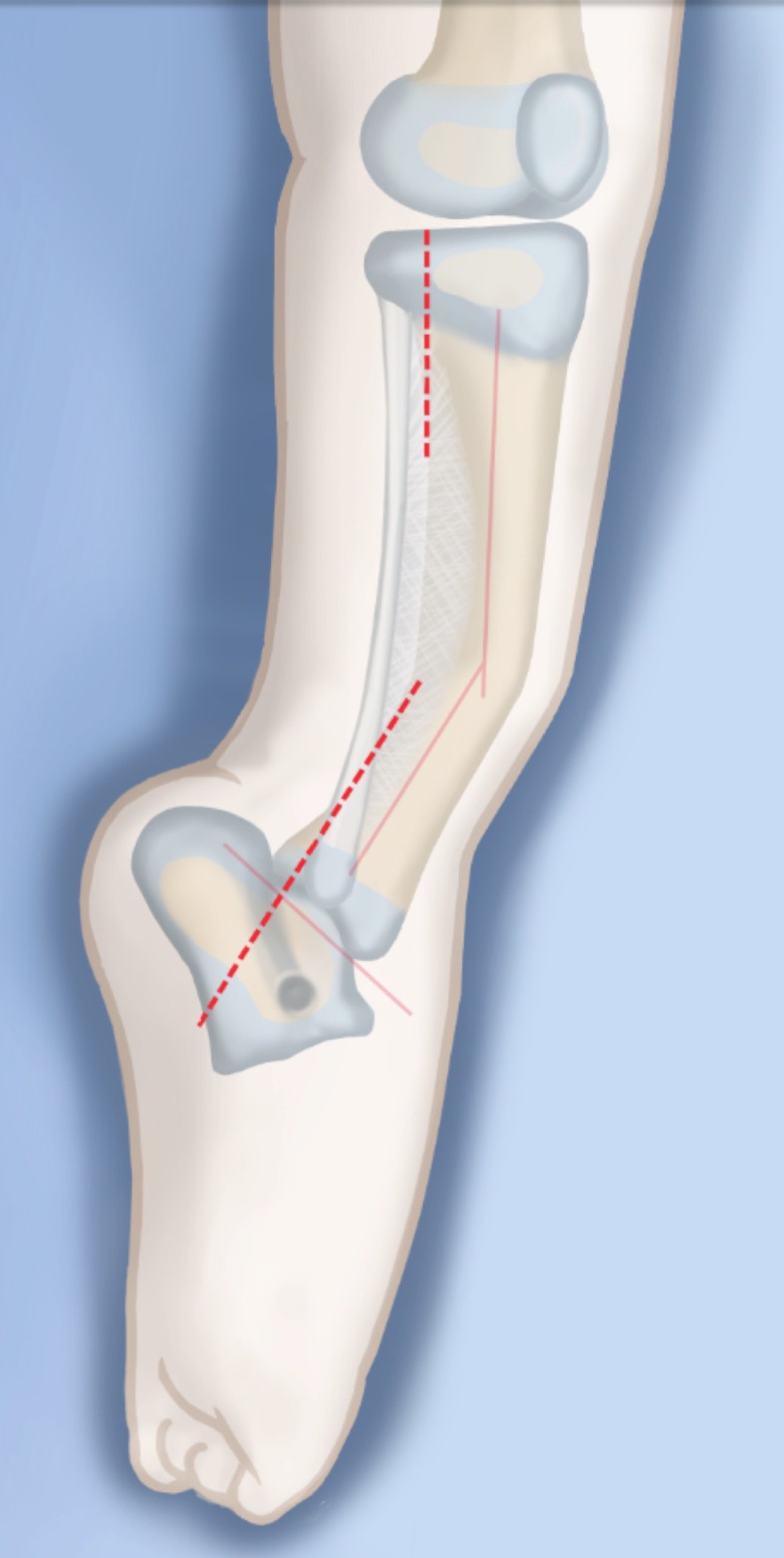
A lateral longitudinal incision is made along the posterior aspect of the tibia. A second incision is made where the neck of the fibula would normally be. Reproduced with permission by the Paley Foundation

**Fig. 20:**
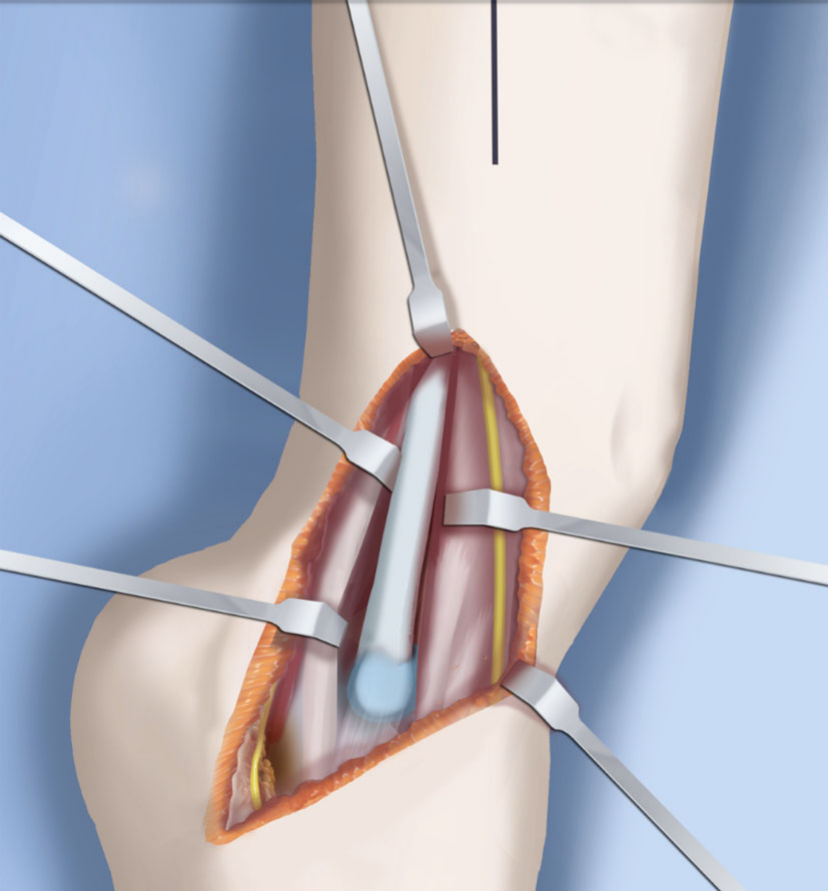
The incision ends at the sural nerve distally. The peroneal sheath is opened. The superficial peroneal nerve is decompressed and protected. The fibular anlage is exposed. Reproduced with permission by the Paley Foundation

**Fig. 21:**
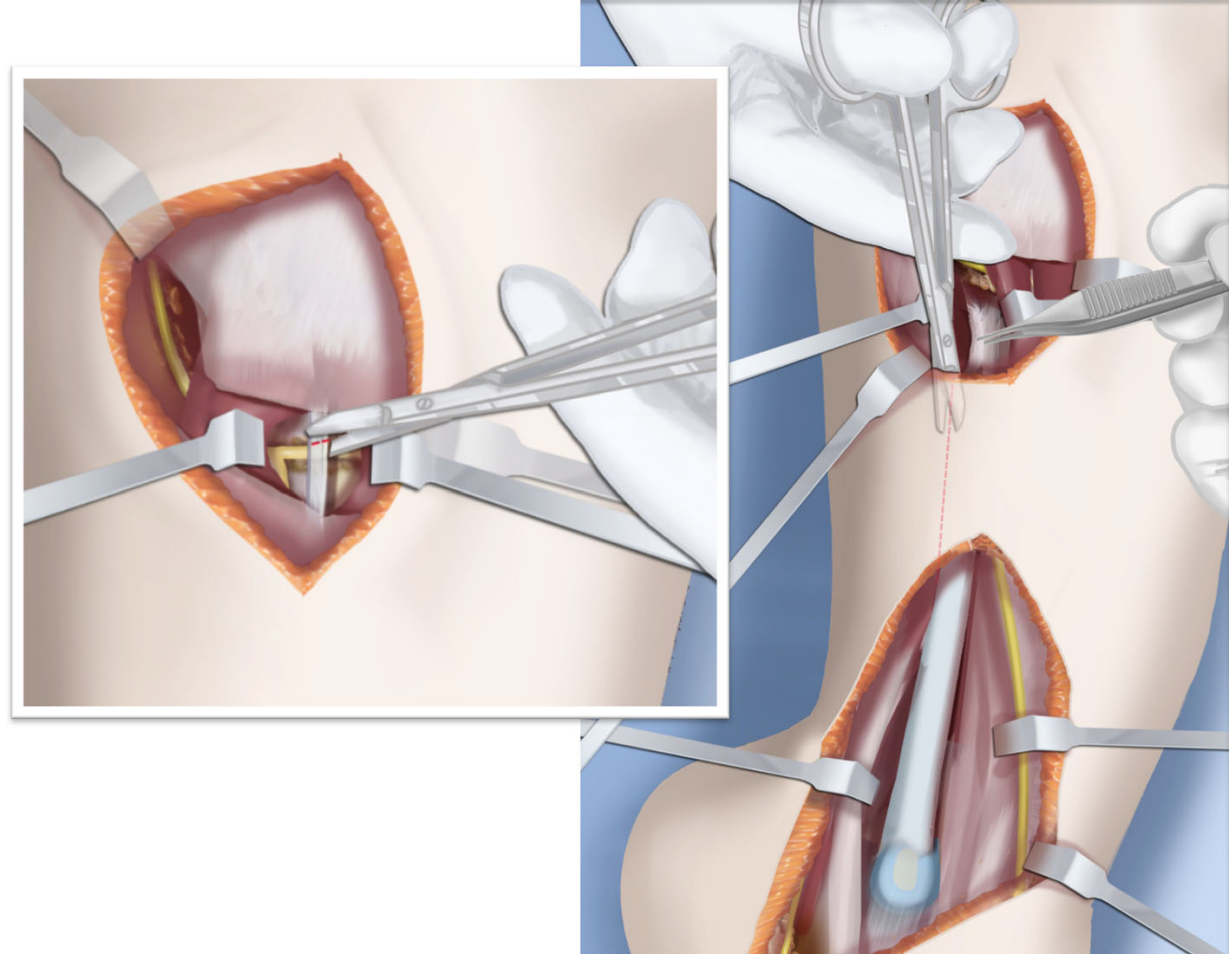
The peroneal nerve is decompressed and the anlage is dissected free proximally and under the skin bridge. Reproduced with permission by the Paley Foundation

**Fig. 22:**
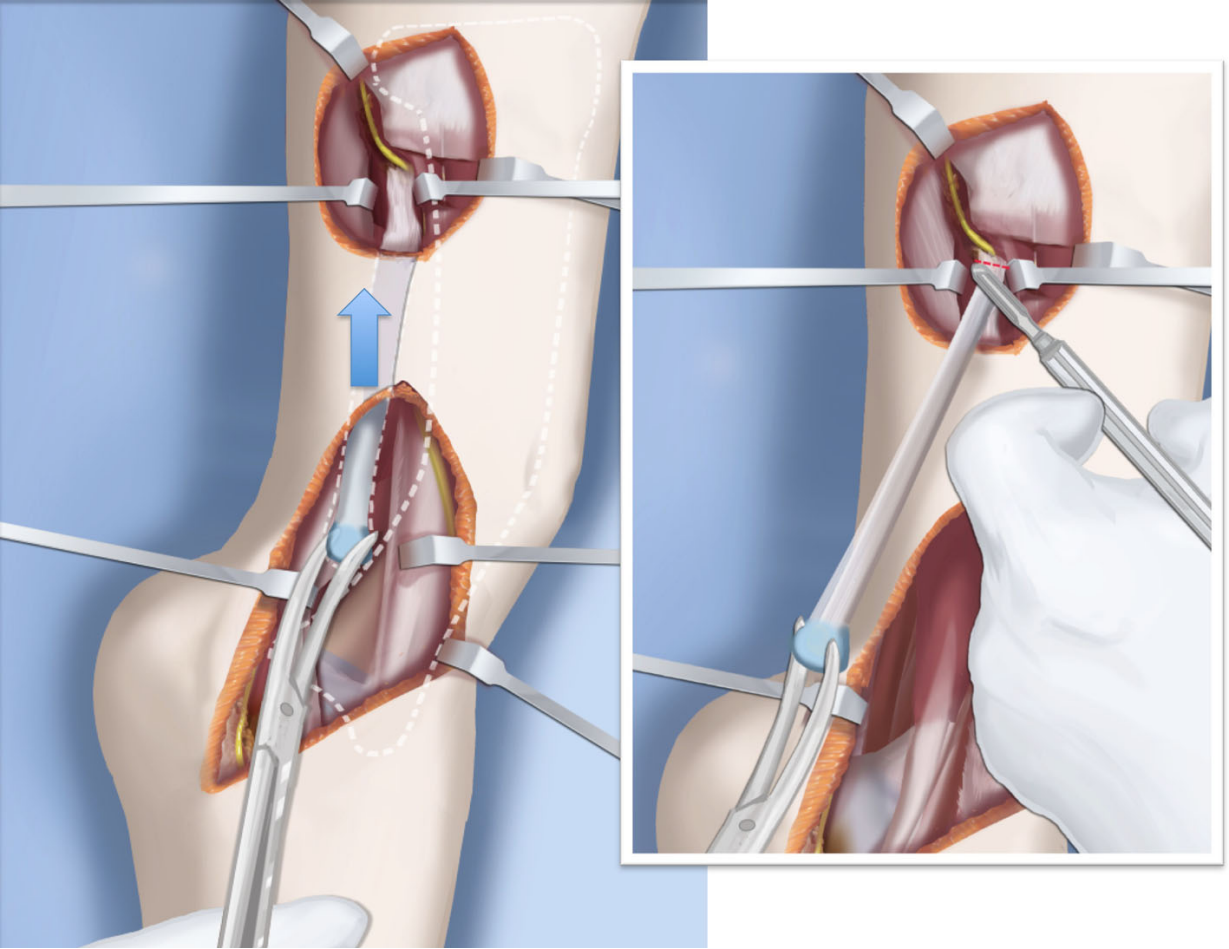
The anlage is resected. Reproduced with permission by the Paley Foundation

**Fig. 23:**
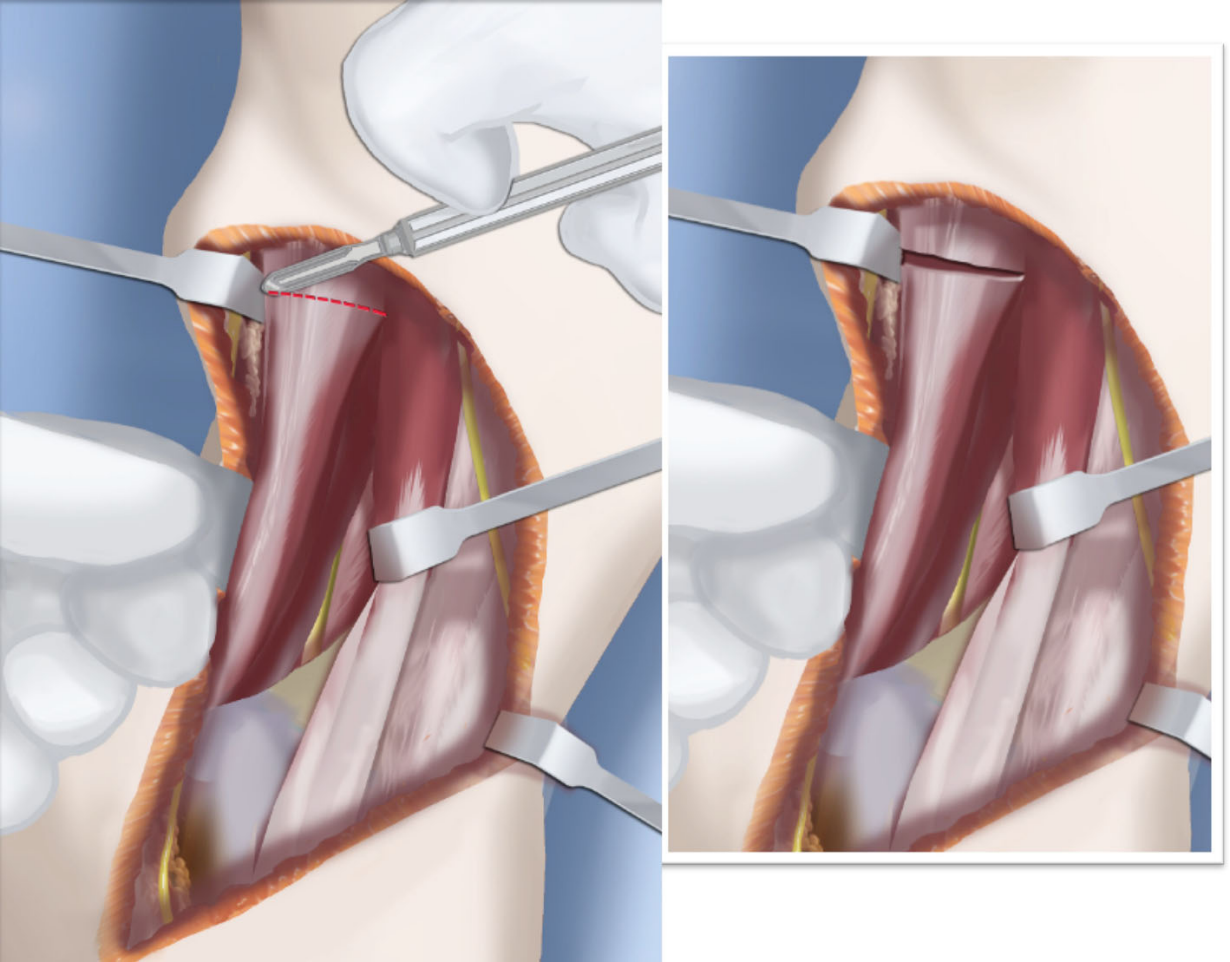
Perform a gastro-soleus recession. Reproduced with permission by the Paley Foundation

**Fig. 24:**
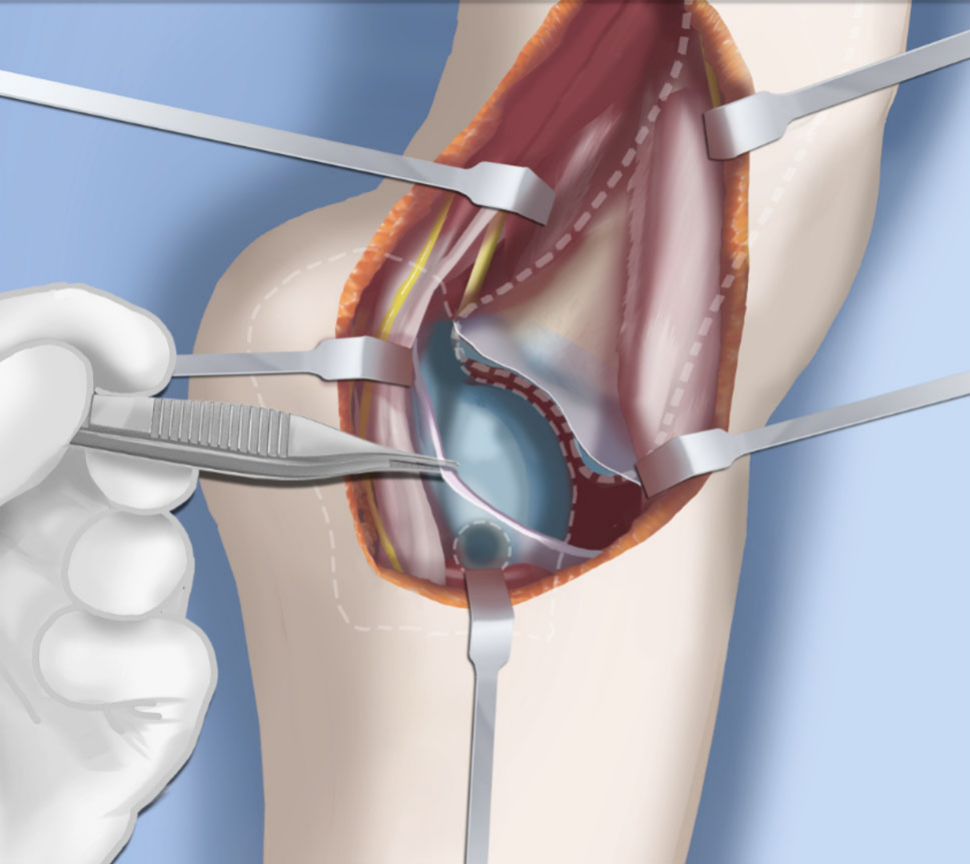
Expose the lateral wall of the talus and calcaneus and perform an ankle capsulotomy. Reproduced with permission by the Paley Foundation

**Fig. 25:**
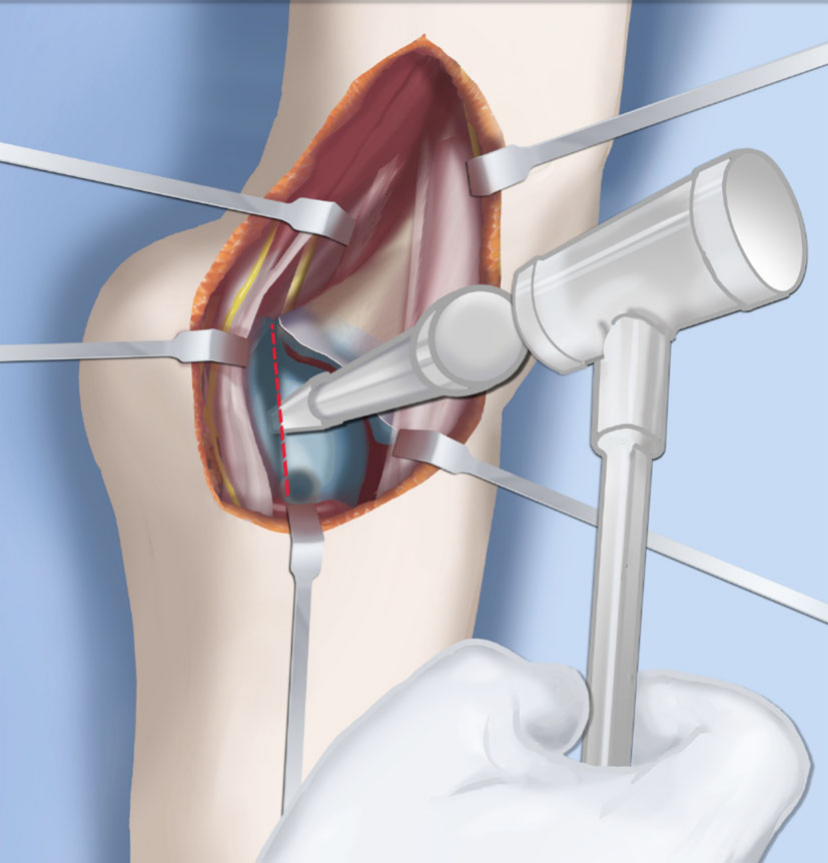
Perform a subtalar osteotomy at a 45° angle, from proximal lateral to distal medial. Reproduced with permission by the Paley Foundation

**Fig. 26:**
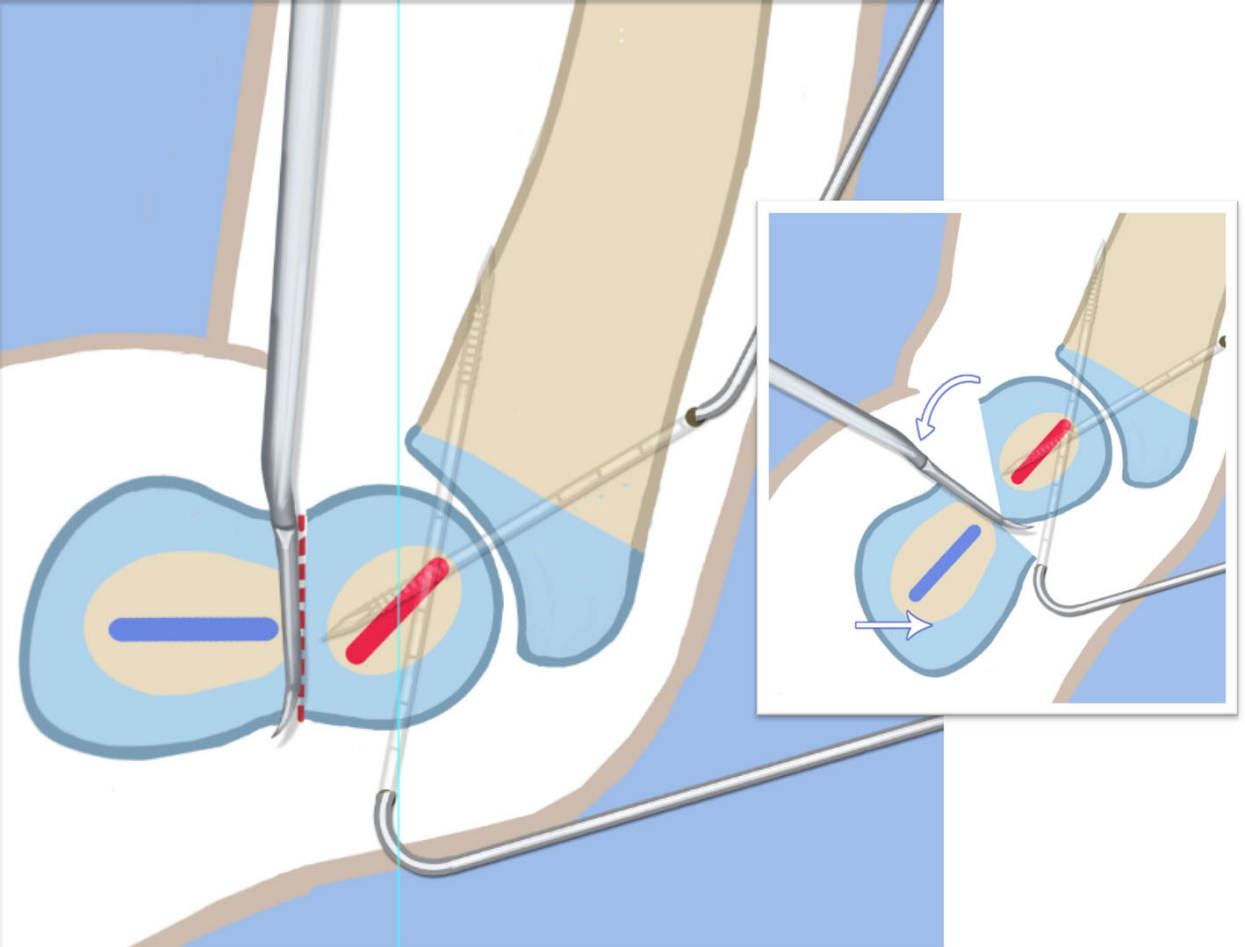
Temporarily fix the talus to the tibia with two wires from the medial side. Lever the subtalar osteotomy using a Hohmann elevator from the lateral side. This corrects valgus and lateral translation. Reproduced with permission by the Paley Foundation

**Fig. 27:**
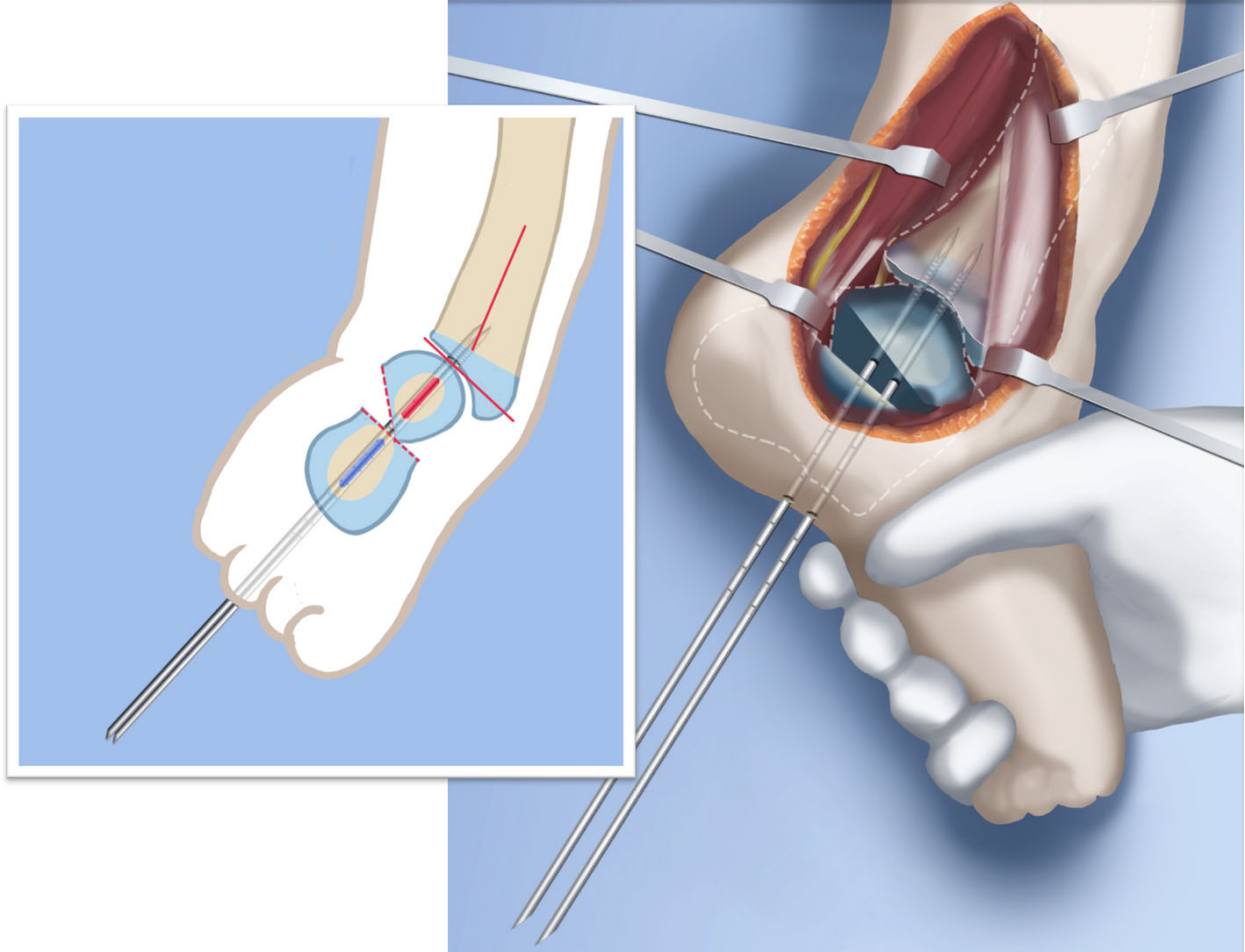
Fix the osteotomy in place with two retrograde wires. These temporarily arthrodese the ankle joint in its neutral position. Reproduced with permission by the Paley Foundation

**Fig. 28:**
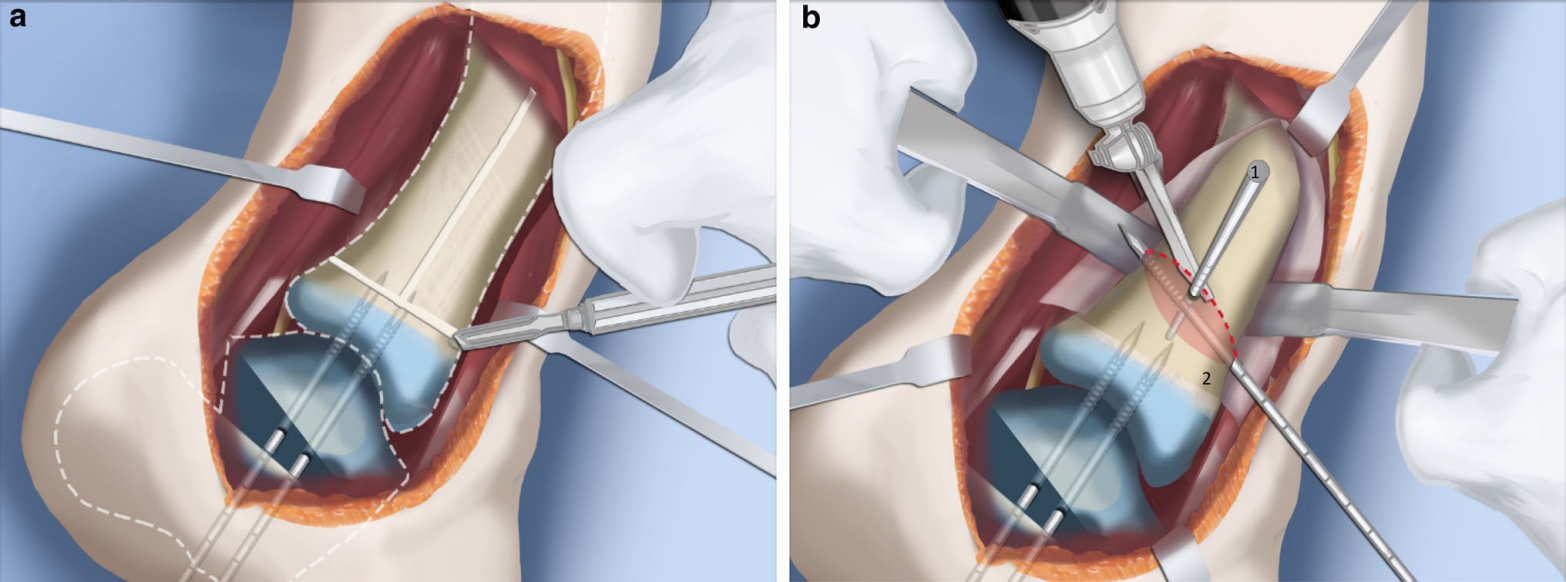
Make a T-incision in the periosteum (**a**). Insert two guide wires parallel to the plantar aspect of the foot in the frontal (*1*) and sagittal (*2*) planes. Make a distal osteotomy parallel to the guide wires using a saw (**b**). Reproduced with permission by the Paley Foundation

**Fig. 29:**
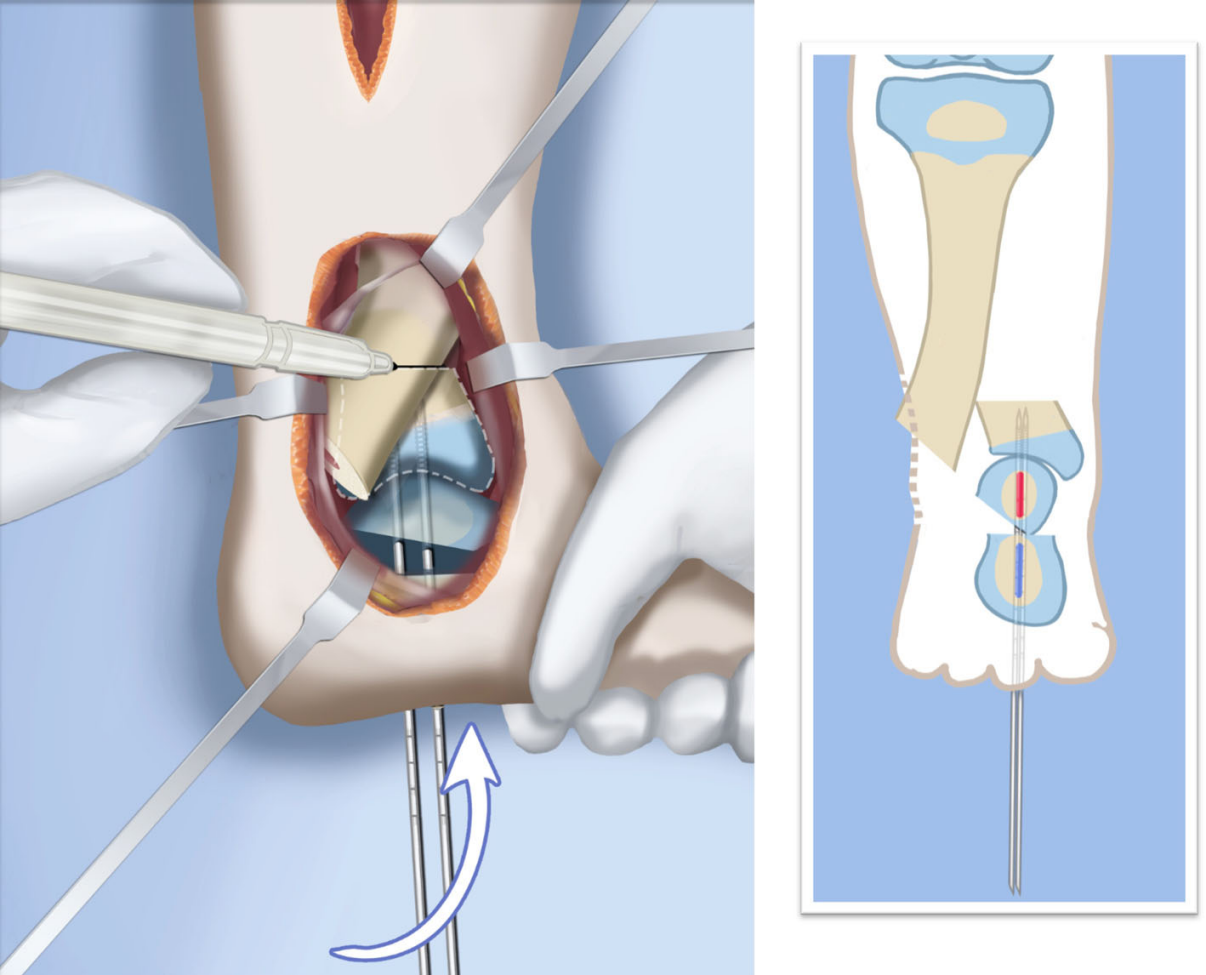
Displace the osteotomy and disengage the bone fragments. Overlap the bone ends. The amount of overlap represents the amount of shortening required to acutely correct the foot. Reproduced with permission by the Paley Foundation

**Fig. 30:**
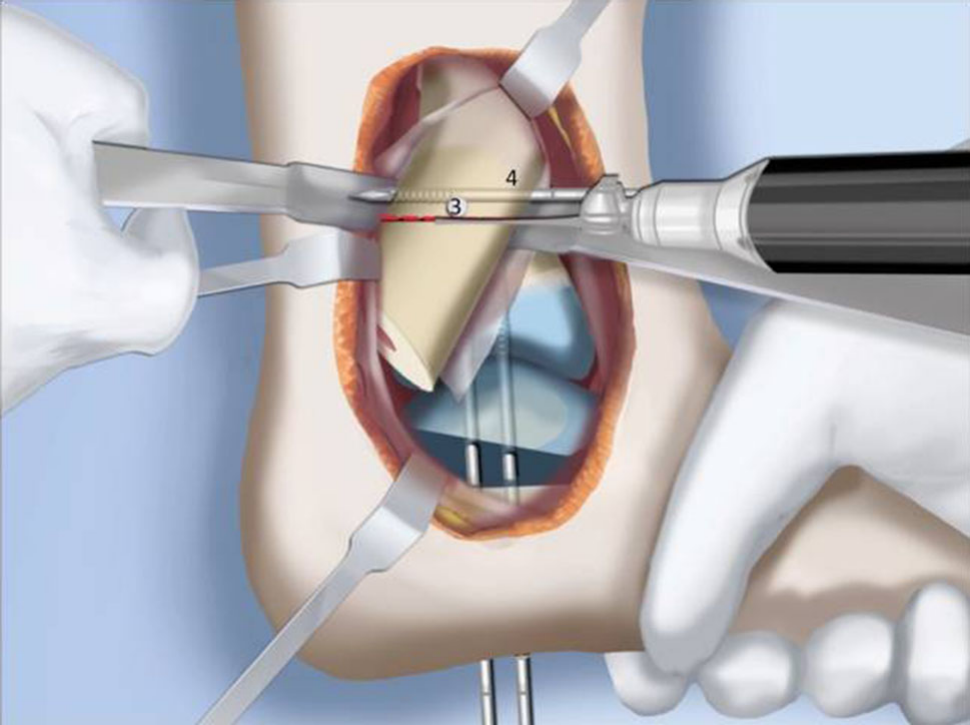
After inserting two guide wires (*3* frontal plane; *4* sagittal plane) inserted perpendicular to the proximal tibia at the level of the overlap, a second osteotomy is performed to resect the bone segment. Reproduced with permission by the Paley Foundation

**Fig. 31:**
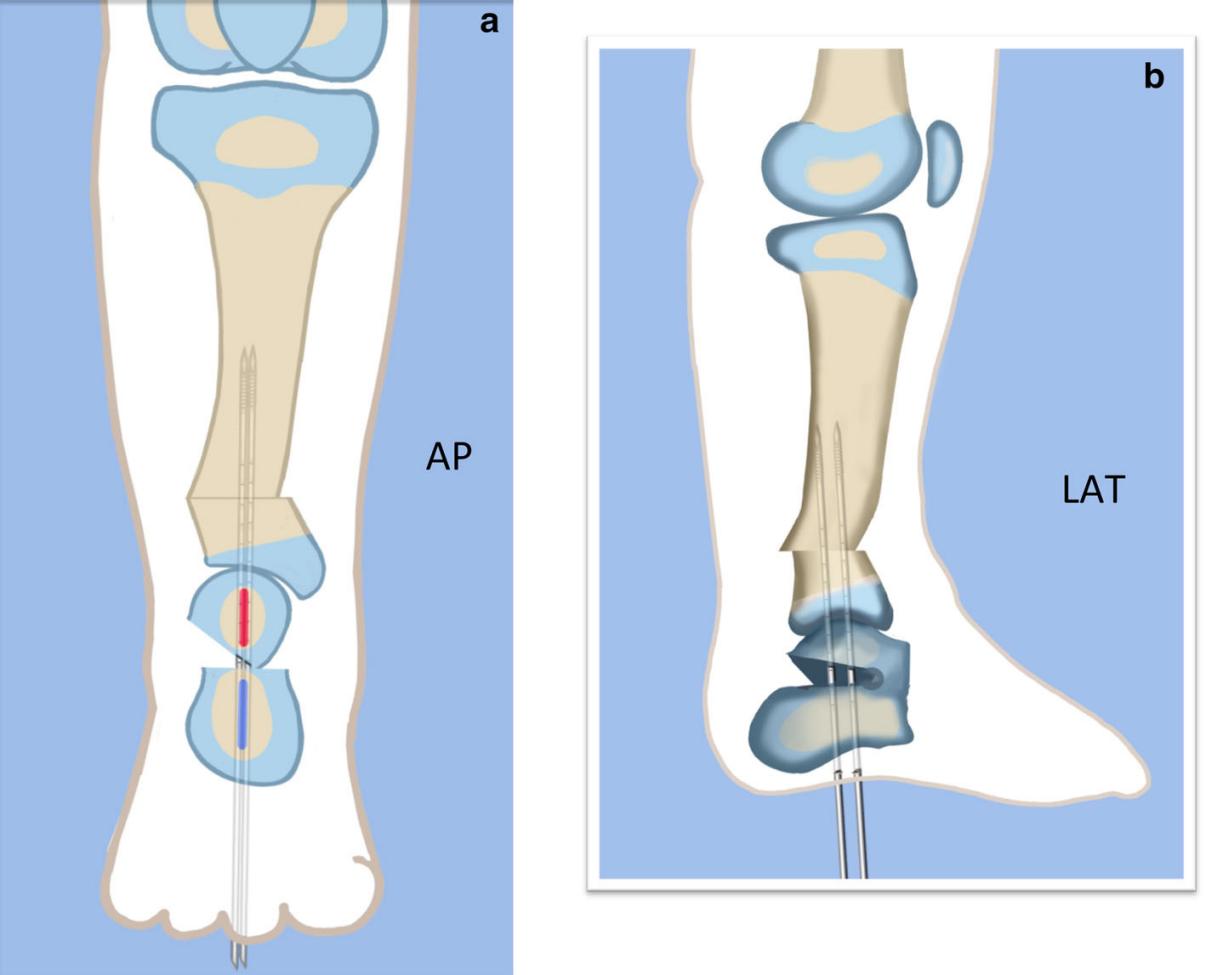
Final alignment on AP (**a**) and LAT (**b**) views. Axial k-wires advanced up the tibia. Reproduced with permission by the Paley Foundation

**Fig. 32:**
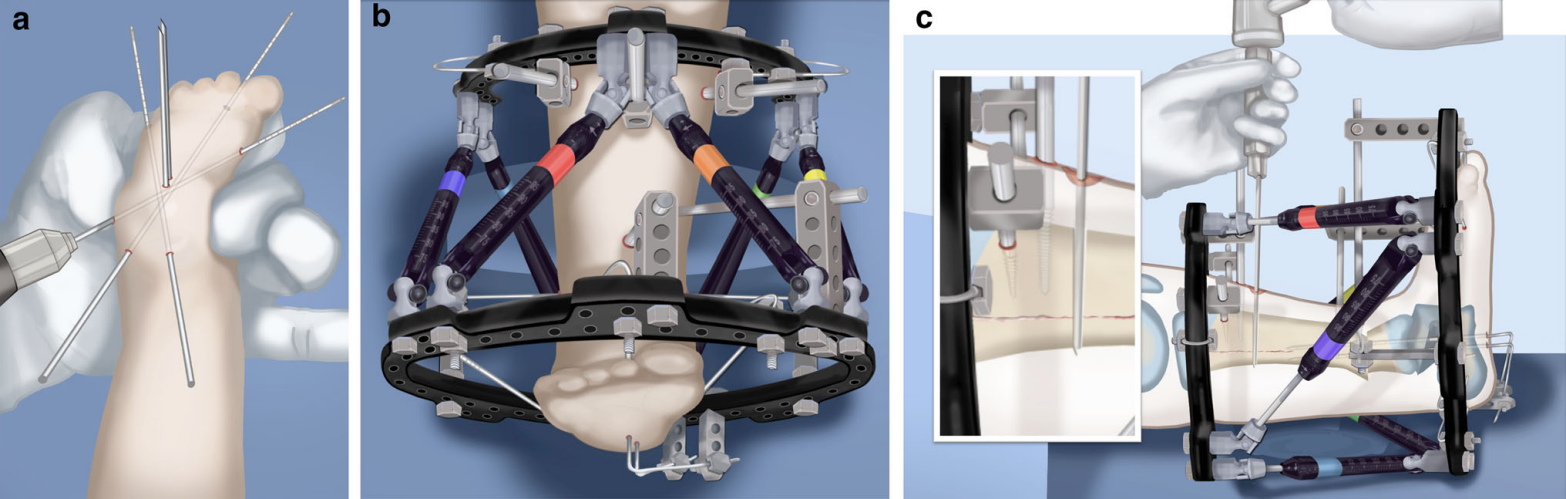
Mount external fixator to tibia and foot. Start by inserting wires in foot parallel to sole (**a**). Apply proximal and distal rings with half pins and wires (**b**). Perform a proximal osteotomy for lengthening (**c**). Reproduced with permission by the Paley Foundation

**Fig. 33:**
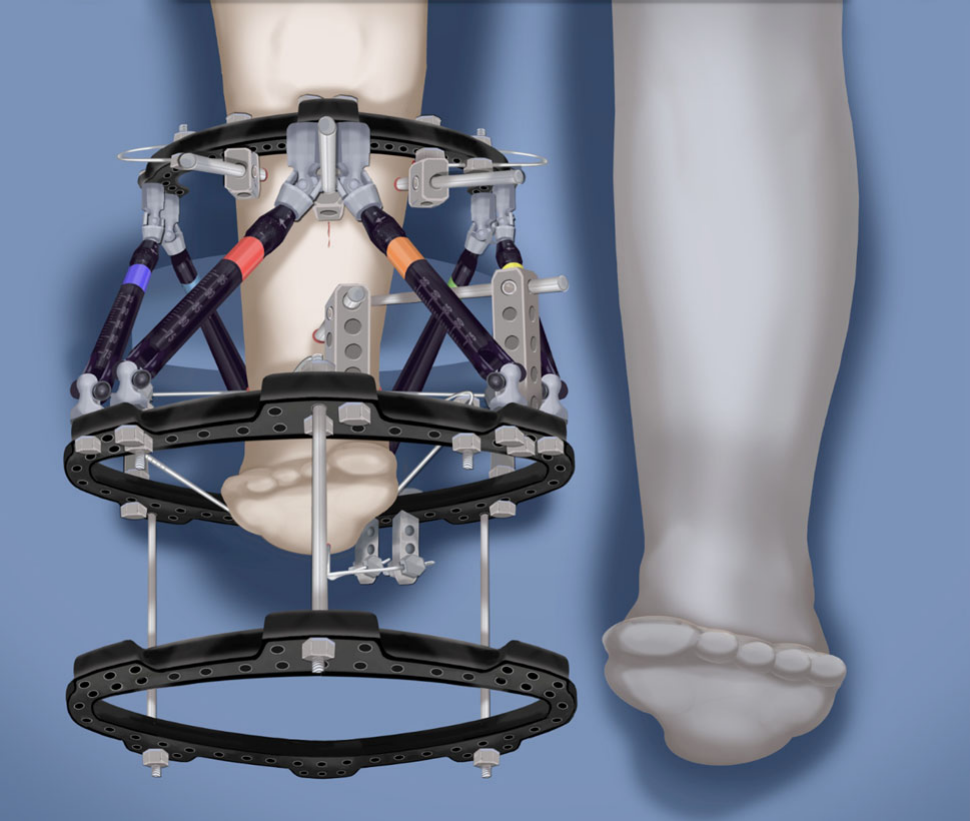
Add a walking ring and secure axial wires to the frame. Reproduced with permission by the Paley Foundation

**Fig. 34:**
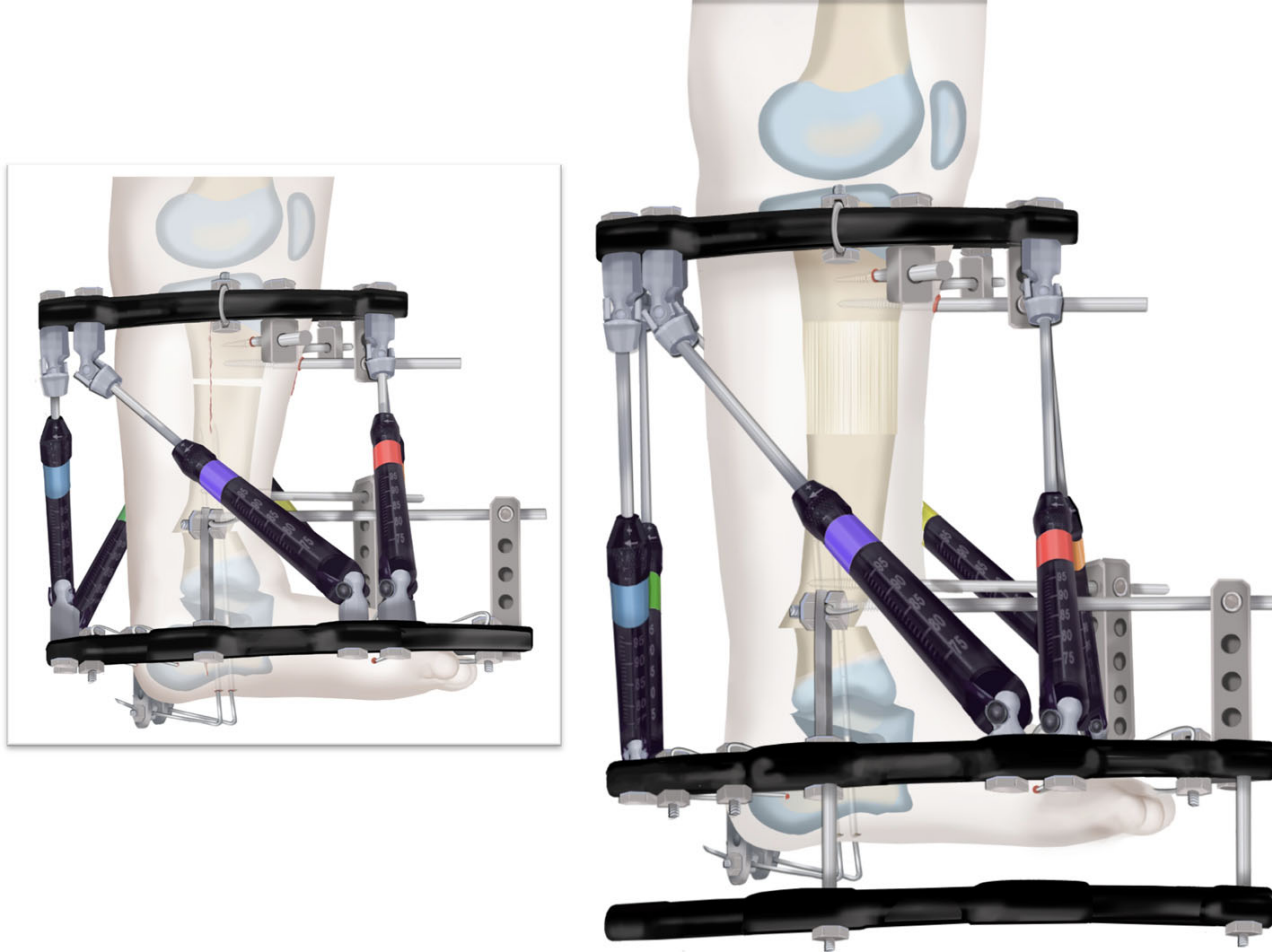
The leg is lengthened. Reproduced with permission by the Paley Foundation

**Fig. 35:**
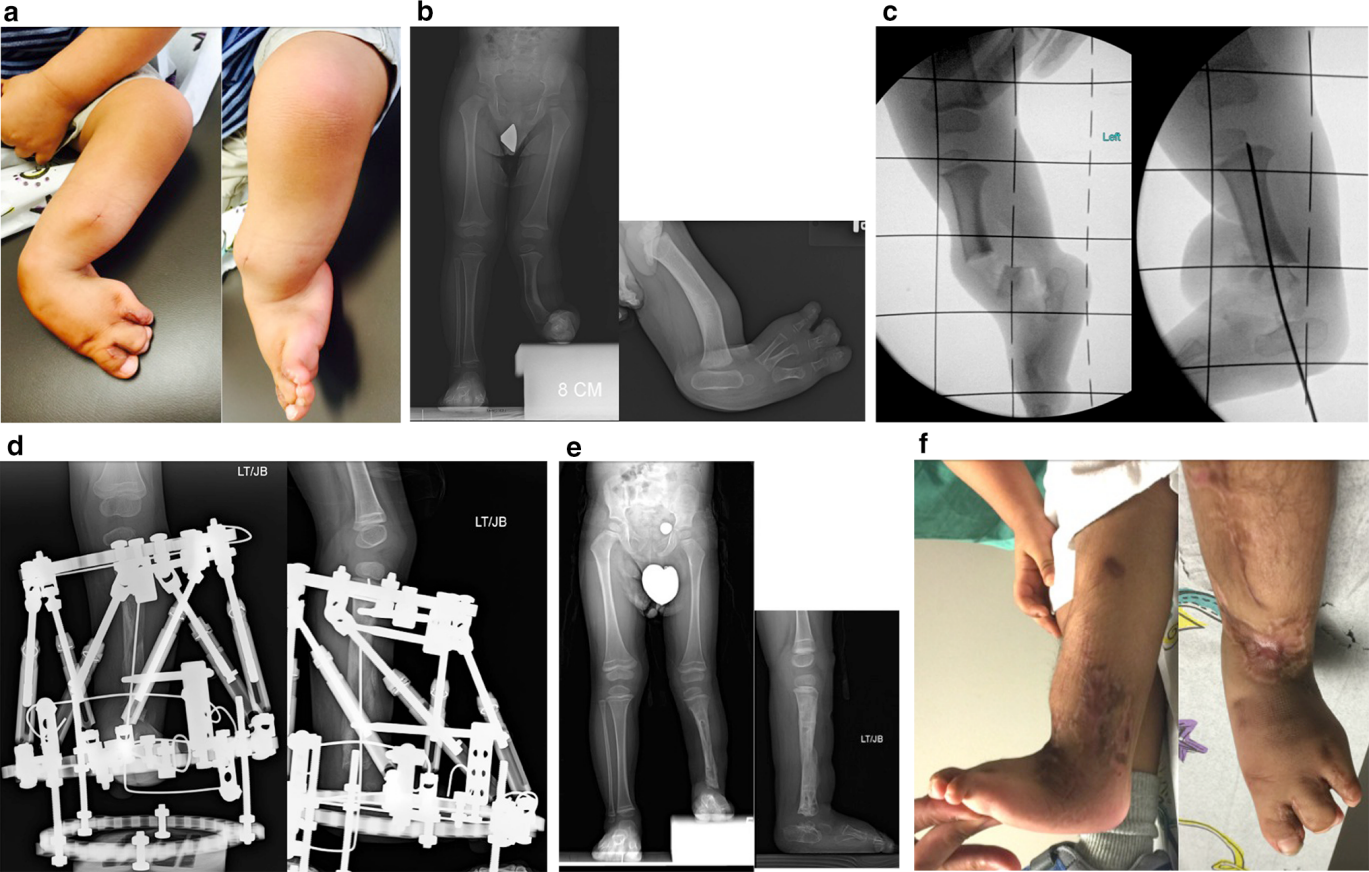
**a** Photographs of foot/leg of 18-month-old-boy with FH, Paley type 3C. The foot is in fixed equino-valgus deformity. He has already had desyndactyalization of his first and third webspaces of the foot at age 12 months. **b** Standing erect legs radiograph showing his leg length discrepancy and lateral radiograph showing the diaphyseal bowing and foot valgus. Note there appears to be only one tarsal bone on the lateral view. He stands on the outer border of his foot. **c** Intraoperative radiographs showing the bone resection (*left*) and foot realigned and pinned in a plantigrade position (*right*). **d** AP and lateral radiographs showing lengthening of 5 cm through a proximal osteotomy using Taylor Spatial Frame apparatus (Smith & Nephew, Memphis, TN). The new bone formation is excellent. **e** AP and lateral radiographs after removal of external fixator. The external fixator was on the leg for 4 months. These show the foot is plantigrade, the diaphyseal angulation has been corrected, the ankle is reoriented and both talus and calcaneus can be seen on the lateral view. The leg length discrepancy is reduced from the preoperative radiograph. **f** Final photographs of foot showing plantigrade foot

The SUPERankle procedure was first developed by the author in 1996 [24]. This procedure achieves a stable plantigrade foot and ankle. It can be combined with lengthening, but it does not have to be. I prefer to perform this procedure between when the patient is between 18 and 24 months of age if it is to be combined with lengthening at the same time. I have performed the SUPERankle as early as 12 months of age, without lengthening. The original Paley SUPERankle procedure involved surgical lengthening of the Achilles and peroneal tendons [16] combined with opening wedge osteotomies of the distal tibia and/or subtalar coalition. While the results of this original SUPERankle procedure were excellent, the author noticed that in long-term followup there was weak push-off strength due to the lengthening of the Achilles tendon. Furthermore, many patients developed a supination midfoot deformity with a dorsal bunion due to overpull of the tibialis anterior from a weak peroneus longus tendon. In 2008, the author modified the procedure to avoid lengthening the Achilles or peroneus longus tendons by shortening the distal tibial osteotomy instead of performing an opening wedge at that level. This newer version of the SUPERankle produced much better functional results with respect to the strength of the gastro-soleus muscles and the peroneal muscles. Push-off strength was conserved, and no supination deformity resulted. The following description is therefore the SUPERankle procedure as currently performed by the author (Figs. 18, 19, 20, 21, 22, 23, 24, 25, 26, 27, 28, 29, 30, 31, 32, 33, 34, 35). While others [16] have suggested separating the lengthening from the SUPERankle procedure, this author sees no advantage to this proposed alternative procedure. The concern that simultaneous lengthening with the SUPERankle could result in increased stiffness of the ankle joint has not been borne out in this author’s experience (Figs. 34, 35). The primary determinant of the ankle range of motion is related to the dysplasia of the ankle joint. The talus in most of these patients has a very limited arc of curvature and a short talar neck. Plantar flexion is limited by impingement with the calcaneus posteriorly, due to the subtalar coalition and the malposition of the calcaneus with the talus. Dorsiflexion is limited by the short neck of the talus and its impingement with the neck due to the lack of talar neck offset (concavity). The less dysplastic the talus is to begin with, the greater the potential range of ankle motion; the more dysplastic the talar anatomy, the greater the limitation of ankle motion. Therefore, whether the SUPERankle is performed with or without lengthening, the range of motion of the ankle is not impacted. The goal of the SUPERankle procedure is to correct the alignment and stability of the ankle joint and foot.

#### Surgical procedure

Step 1: Under tourniquet control, make a lateral longitudinal incision in line with the posterior border of the tibia from the level of the diaphyseal bend in the tibia (when present) to the level of the sural nerve. Identify and dissect free the sural nerve to avoid cutting it (Figs. 19, 20).
Step 2: Incise the lateral fascia from distal to proximal. Look out for the superficial peroneal nerve as it exits the fascia. Decompress this nerve proximal and distal to the level it exits the fascia (Fig. 20).
Step 3: Identify the peroneal tendon(s). If there are two tendons, mobilize them and only lengthen the brevis and never the longus. If there is one conjoint tendon present, and it appears to be a brevis tendon (attached to lateral calcaneus or to lateral side of foot but not extending to first metatarsal), then lengthen this tendon in a Z-fashion. If it is the longus tendon, free it from its sheath and allow it to move anteriorly outside of its sheath without cutting it. This effectively lengthens the tendon without weakening it (Fig. 21).
Step 4: Identify the cartilaginous and fibrous fibular anlage. This is almost always present. Dissect its borders free anteriorly and posteriorly working from distal to proximal. Separate it from the adjacent calcaneus. In some cases it may actually be fused to the calcaneus. In these cases, cut through the cartilage bridge connecting it to the calcaneus (Fig. 20).
Step 5: Make a second small longitudinal incision at the level of the proximal tibia in line with where the fibular neck would have been (Fig. 21).
Step 6: Palpate and find the peroneal nerve just under the fascia and follow it to the peroneal fascia. Incise the peroneal fascia over the muscle and then go retrograde to decompress the nerve by cutting the overlying fascial band that is entrapping the common peroneal nerve. Extend the transverse fasciotomy towards the tibia. Identify the intermuscular septum between the anterior and lateral compartments. Separate the muscles from either side of it and then cut the septum all the way down to where it passes over the deep peroneal nerve (Fig. 21).
Step 7: Find the separation between the gastrocnemius muscle and the peroneal muscles. Separate this interval distal to the nerve to find the proximal fibrous fibular anlage. Dissect distally along this anlage (Fig. 21).
Step 8: Work from both ends to free the anlage from the surrounding muscles. Free the distal end of the anlage from the calcaneus and then fold it over to pass it out the proximal incision by tunneling between the two incisions. Be careful not to damage the superficial peroneal nerve. Pull the anlage out proximally and then resect it just distal to the nerve (Fig. 22).
Step 9: Return to the distal incision and identify the flexor hallucis longus (wiggle the big toe). Posterior to this muscle, lie the posterior tibial nerve, artery and veins. They lie on the medial border of the soleus muscle belly.
Step 10: Identify the lateral border of the soleus muscle and find its tendinous apponeurosis. Perform a tranverse recession of the gastro-soleus aponeurosis [26]. Watch out for theneurovascular structures on the medial border (Fig 23).
Step 11: Follow the posterior tibial neurovascular bundle to the medial wall of the calcaneus and decompress it from the calcaneus.
Step 12: Clean the lateral wall of the calcaneus by reflecting the extensor digitorum brevis from its surface from posterior to anterior. Identify the sinus tarsi and the posterior border of the calcaneus where it lies against the tibia (Fig. 24).
Step 13: Identify and incise the lateral capsule of the tibio-talar (ankle) joint. Examine its shape to determine if it is round and whether its curvature will limit ankle motion. Identify the junction between the talus and calcaneus posteriorly. Note that a subtalar coalition is present. Use an osteotome to cut through this coalition at a 45° angle from proximal lateral to distal medial. Start this cut at the talo-calcaneal junction at the posterior edge of the ankle joint. The cut extends to the sinus tarsi. After the cut is completed, carefully distract it to visualize the posterior tibial neurovascular bundle. Use this additional exposure to further decompress the nerve (Fig. 25).
Step 14: Pin the talus to the tibia with one medial antegrade wire and one medial retrograde wire. Allow the talus to sit in its undisturbed, uncorrected position before pinning it (Fig. 26).
Step 15a: Now displace the subtalar osteotomy by levering the calcaneus to move distal and medial using a small Hohmann elevator. This takes time and patience to get the calcaneus to move. It should slide medial-distally and not wedge open on the lateral side. Its cut surfaces should remain in contact. Once the calcaneus is medialized, varusized and distalized, it should be pinned with two retrograde wires through the foot. Advance these pins from the calcaneus to the talus across the ankle joint to the level of the distal tibial physis. Remove the two temporary medial pins crossing the ankle joint (Fig. 26).
Step 15b: If there is an abductus foot deformity this is almost always related to an associated calcaneocuboid coalition tethering the foot into abductus. This coalition will block the correction of the subtalar osteotomy. Therefore an osteotomy or chondrotomy of the coalition must be performed prior to displacing the subtalar coalition osteotomy. The subtalar coalition malunion can then be corrected and pinned as described above. To hold the abductus foot correction, insert a posterior to anterior wires in the plane of the sole of the foot from the calcaneus, across to the cuboid and anteriorly to exit the foot. This wire can later be incorporated into the external fixation of the foot by fixing it to the foot ring.
Step 16: Insert supramalleolar tibial guide wires in the frontal and sagittal planes parallel to the plantar aspect of the foot as distal as possible without crossing the distal tibial physis (Fig. 28).
Step 17: Osteotomize the distal tibia parallel to these guide wires (Fig. 28).
Step 18: Shift the distal segment medially and overlap the tibial bone ends. Mark the level of the overlap. Insert guide wires at the level of the overlap which is the level of the second osteotomy. The second osteotomy should be made perpendicular to the axis of the proximal diaphysis of the tibia. If there is a diaphyseal procurvatum-valgus angulation the second osteotomy may be at or distal to the apex of this deformity. Use a saw to osteotomize the tibia again. This second osteotomy is for straightening and shortening the tibia. A trapezoidal shaped piece of bone is resected (Figs. 29, 30).
Step 19: After the guide wires and bone segment are removed, the tibia can be realigned and shortened. The two distal axial wires can be advanced up the tibia and if the cuts were performed correctly the foot will be plantigrade. The skin over the anterior distal tibia can be dissected free off the tethering bone to avoid creating a skin crease anteriorly (Fig. 31).
Step 20: The tourniquet can now be removed and the wounds closed over a drain. There are no tendons to repair.
Step 21: If lengthening is to be performed at the same surgery, an arthrogram is carried out at the knee joint. This identifies the knee joint line. The proximal external fixator wire is inserted parallel to this line just distal to the physis. The proximal 2/3 ring of a computer-dependent external fixator is applied to this wire. A half pin is inserted into the anterior tibia and fixed to the ring with a cube (Fig. 32).
Step 22: The foot is fixed with three wires; the first calcaneal wire enters postero-midline in the calcaneus to exit between the first and second toes. The next two crossed wires enter postero-lateral and posteromedial to this first wire to exit antero-medial and antero-lateral, respectively. These three wires are parallel to the sole of the foot. They are fixed and tensioned to a full ring which passes circumferentially around the foot (Fig. 32).
Step 23: Six struts are connected between the two rings. Once the struts are in place the remainder of the fixation is added. This includes one anteromedial and anterolateral half pin proximally and two half pins and a wire in the tibia distally (Fig. 32).
Step 24: The final step is to perform the osteotomy for lengthening. This is performed anywhere in the proximal tibia distal to the proximal pins. Since the foot is now plantigrade, there is no need to perform this osteotomy at an apex of angulation. The axial wires are backed out until the osteotomy is completed. And then re-advanced across the osteotomy site (Fig. 32).
Step 25: Reference shots and planning is carried out after surgery. A schedule for lengthening is given to the patient. A walking ring is added below the distal ring (Fig. 33).
Step 26: If a valgus deformity of the knee exists from the femur, insert a hemi-epiphsiodesis plate (e.g. 8 plate; Orthofix, McKinney, TX).

It should be noted that:

- The above description is the SUPERankle variety used for Paley type 3C, which is the most common type 3 (Fig. 18). For type 3A the tibial osteotomy with shortening alone can be performed.
- For type 3B1 when a fibula is present and is articulating with the calcaneus, perform the SHORDT combined with a subtalar osteotomy.
- For type 3B2 only a subtalar osteotomy is performed.
- For type 4, carry out the procedure as for type 3C with a closing wedge osteotomy of the subtalar coalition, medializing and tilting the talus into valgus instead of varus since there is a varus malunion as opposed to the valgus malunion seen in types 3B and C.

In summary, knowing the Paley classification type of FH determines very specifically the type of osteotomy that should be performed. The SHORDT and SUPERankle procedures are applied according to the specific pathoanatomy of the foot and ankle deformities.

### Knee valgus deformity

Most cases of FH have associated genu valgum secondary to distal femoral and/or proximal tibial valgus deformity. Valgus of the knee can negatively impact the foot. Since there is usually no subtalar joint present, genu valgum cannot be compensated by a mobile subtalar joint. The ankle joint, which is often a ball and socket type, cannot compensate for a valgus knee since it usually has valgus instability (dynamic valgus). After foot deformity correction with the SHORDT or SUPERankle procedure, knee valgus can promote recurrent ankle deformity. It is therefore important to identify and treat the knee valgus to improve the results of the foot correction and to help prevent recurrent ankle valgus. To objectively identify the level of the knee valgus, the lateral distal femoral angle (LDFA) and medial proximal tibia angle (MPTA) should both be measured off of the distal femoral joint line. In young children this line is difficult to see since most of the distal epiphysis is not ossified. It may be necessary to do a knee arthrogram to measure the LDFA and MPTA accurately. If the valgus is from the femur, hemiepiphysiodesis of the distal femur can be carried out using a screw-plate device at the time of the ankle surgery. If the deformity is from the tibia, and if tibial lengthening is carried out, then the deformity can be corrected through the lengthening osteotomy of the proximal tibia. If the tibia is not being lengthened a hemi-epiphysiodesis device can be applied to the proximal tibial physis.

Progressive genu valgum after lengthening is another cause of valgus in patients with fibular hemimelia. Paley et al. found that 75% of patients younger than 12 years and all patients younger than 4 years developed this problem. The deformity recurs through the proximal tibia. The origin is unclear but follows a pattern similar to that seen with the Cozen phenomenon [27] after proximal tibial metaphyseal fractures. In FH, the progressive tibial valgus may be related to the lack of growth by the fibula or may be due to soft tissue tethers on the lateral side by the fibular anlage. It may also be related to the tendency for the proximal tibial epiphysis to ossify medially but not laterally, thereby creating an intra-articular component. Intentionally deforming the tibia into 10–15° of varus at the end of the lengthening compensates for the expected rebound valgus. Another approach is to insert a hemi-epiphysiodesis plate at the end of the lengthening. A similar valgus tendency is observed with progressive valgus deformity in children with FH after amputation [28]. In contrast to the post lengthening tibial valgus, femoral valgus associated with FH is nonprogressive [29]. Femoral valgus may contribute to valgus overload, which may be a factor for valgus rebound in the tibia. Distal femoral hemi-epiphysiodesis can be done at the time of the index lengthening procedure. Complete fibrous anlage resection may reduce the frequency and degree of rebound but has not eliminated the problem.

Growth inhibition has been reported after tibial lengthening for FH [30]. Sharma et al. [30] concluded that this is related to complete fibular aplasia. Most of the cases presented by Sharma et al. were treated with double-level or combined femur and tibial lengthening without soft tissue release. Hope et al. [31], who used only single-level lengthening, could not demonstrate any growth inhibition. Sabharwal et al. [21] showed that growth inhibition occurred only if there had been a second tibial lengthening performed within a year of the first lengthening.

### Knee ligament reconstruction

Most patients with FH have some knee ligament deficiency of the cruciate ligaments. If this instability is symptomatic or if the knee remains subluxed anteriorly in full extension, knee ligament reconstruction with the SUPERknee procedure [32–34] may be required together with the treatment of the ankle or at a separate time. Unlike femoral lengthening for congenital femoral deficiency, knee reconstruction or stabilization of the knee are not required in order to proceed with tibial lengthening.

### Toe and metatarsal surgery

Many patients with FH are missing one or more toes. Some surgeons consider absence of two or more metatarsals an indication for amputation [13]. My results do not support this [21, 35, 36]. As long as the foot is plantigrade, the foot in FH is very functional even with one, two, three or four rays.

Hallux varus, syndactaly and conjoint delta first metatarsals are the most common toe deformities associated with FH that benefit from surgical treatment of the toes. Syndactaly of the first to second toes is easily treated by release and skin grafting. Syndactaly between the middle toes does not need to be separated. Hallux varus is always associated with a short bracket (delta) first metatarsal. In most cases this is a conjoint metatarsal (fusion of first and second metatarsal) associated with syndactaly of the first and second toes. The treatment for this requires separation of the syndactaly combined with splitting of the conjoint metatarsal into two parts and reorienting of this osteotomy to realign the first metatarso-phalangeal joint surface.

### Femoral lengthening

Femoral lengthening can be combined with the tibial lengthening at the same time or at a separate time to treat concomitant shortening of the femur. Simultaneous femur and tibia lengthening with external fixation is used when the femur and tibia shortening is of significant magnitude. In such cases, it is not unusual to perform the SUPERankle procedure with application of the external fixator for lengthening tibia and femur. A discussion of femoral lengthening is beyond the scope of this article, but for further information the reader is referred to published studies [32–34]. If femoral lengthening is considered, it is factored into the surgical life plan discussed previously. Obviously, simultaneous femoral and tibial lengthening can yield much larger amounts of lengthening in one treatment than tibia lengthening alone. For example, simultaneous 5.0-cm femoral and 5.0-cm tibia lengthening together take a total of 5 months of external fixation, and isolated tibia lengthening of 5.0 cm also takes a total of 5 months of external fixation. Therefore, in the first example combined femoral and tibia lengthening achieve 10.0 cm (4 in.) of leg length equalization compared to only 5.0 cm (2 in.) when only the tibia is lengthened. While tibial lengthening alone requires daily physical therapy, combined femur and tibial lengthening mandates strict lengthening-specific physical therapy [33]. There is no indication to do femoral lengthening in the absence of femoral discrepancy. The advent of internal lengthening methods makes femoral lengthening as a separate procedure much easier.

## Results

The results of SUPERankle reconstruction versus amputation were studied by Paley et al. [36]. These authors compared 20 children treated by primary amputation at one institution with 22 children treated using the SUPERankle reconstruction with limb lengthening at another institution. Both patients and parents completed psychosocial, quality of life (QoL) and satisfaction surveys. All patients underwent instrumented gait analysis and a timed 50-yard dash. At the time of evaluation, the average patient age was 9 (range 5–15) years. The main difference between the groups was that families of children treated by amputation had lower economic and educational levels and were more ethnically diverse that those of the limb reconstruction group. Scores on psychosocial and QoL surveys tended to be commensurate with those from healthy patient populations in both groups. Parents of males treated by amputation perceived a lower school-related QoL for their child, a finding possibly explainable by socio-economic and ethnic differences between the groups. All patients and parents reported satisfaction with treatment method selected and would select the same treatment method again. There were no statistically significant differences in average performance in gait analysis or timed 50-yard dash. Using standardized evaluation tools, both groups showed comparable documented psychosocial adjustment, QoL and physical function. The limb lengthening group will require additional lengthening and/or epiphysiodesis to complete leg length equalization.

## Discussion

### Lengthening reconstruction surgery versus amputation

Amputation remains the most common option presented to parents with children who are born with FH. Why is amputation offered as the main treatment option? Performing an amputation at the level of the ankle joint (Syme’s amputation) gives a nice round stump with the heel pad as a weightbearing surface. That combined with modern prosthetics leads to unrestricted excellent function. As we all saw demonstrated in the 2012 London Olympics, amputees, and even bilateral below-the-knee amputees such as Oscar Pistorius, fitted with advanced prosthetics can even compete at the highest level. There is no question that a patient with FH who undergoes a Syme’s amputation and good prosthetic fitting and who has access to a technologically advanced prosthesis and prosthetic care on a regular basis (most children need a new prosthesis each year) will function normally for almost any activity. It is not uncommon to see video clips of children skateboarding, rock-climbing and performing individual and team sports following a below-knee amputation.

Nevertheless, if an amputation could be avoided and the foot and ankle and leg reconstructed to nearly normal function comparable to that afforded by a below-the-knee prosthetic, most parents and most individuals will choose to have the reconstruction. I do not think anybody wants to give up their foot or ankle unless there are no good alternatives.

When pediatric orthopedic surgeons are asked if they would amputate the foot if all that was wrong with the leg was a foot or ankle deformity such as club foot or many other childhood foot deformities, the answer is universally “no”. Despite this, the results of some clubfoot treatments leave the child with chronic pain and a stiff deformed foot that might be better treated by amputation and prosthetic fitting. When pediatric orthopedic surgeons are asked if they would amputate the leg of a child with no foot deformity and just a leg length discrepancy, the answer is almost universally “no”. When pediatric orthopedic surgeons are asked if they will amputate the leg of a child with a combination of foot deformity and a leg length discrepancy, the answer is frequently “yes”. The logic of this does not follow since for a foot deformity the recommendation is to correct the foot and for a leg length discrepancy the recommendation is to lengthen the leg; therefore, should not the recommendation for a foot deformity with a leg length discrepancy be to correct the foot deformity and lengthen the leg?

Most authors agree that lengthening is the preferred treatment for patients with mild to moderate leg length discrepancy with mild foot deformities (Paley types 1 and 2). The controversial cases are those that include more severe foot deformities (Paley types 3 and 4) and greater leg length discrepancies due to more severe tibial growth inhibition or combined femoral and tibial discrepancy. Syme’s or Boyd amputation has been the conventional recommendation for these more severe cases [37]. The justification for amputation for the more severe cases has been the failure of most surgeons to obtain satisfactory results after limb lengthening [38–40]. No one would dispute that amputation with prosthetic fitting requires fewer surgical interventions and fewer days of hospitalization and is associated with a lower complication rate. Furthermore, no one would dispute that with the availability of modern prosthetics, limb length equalization with excellent function can be achieved reliably in patients who have undergone Syme’s or Boyd amputation [28, 37, 41]. This does not prove, however, that the best treatment for severe cases of FH is amputation with prosthetic fitting. Excellent function could also be obtained if amputation and prosthetic fitting were used to treat clubfoot, ankle arthritis or other disabling foot conditions. This is a testimony to the excellence of modern prosthetics and nothing more.

The challenge, therefore, is not to compare the function achieved in cases of Syme’s or Boyd amputation with that achieved in cases of lengthening—but rather to improve the results of lengthening and foot reconstruction in FH [42]. Why are the results that are reported by many authors so poor [42]? Is it because these cases are unreconstructable or is it because of fundamental errors in the treatment strategy used? An analysis of the unsatisfactory results reported in different series in the literature [36–38, 42] makes it clear that the overriding factor associated with poor results is recurrent or residual foot and tibial deformities—and not the inability to obtain equalization of limb length. The few series in which good results were obtained, even in severe cases of FH, reported that the final result was a stable plantigrade foot [43–46]. The total amount of discrepancy can always be equalized by serial moderate-sized lengthenings rather than by one very large lengthening. The foot deformity can be treated by various methods, including soft tissue and bone procedures. If these fail, ankle arthrodesis is a very successful way of permanently stabilizing the foot [43, 45]. It is clear that ankle arthrodesis should not be the indication for amputation. Therefore, because the worst-case analysis in stabilizing and correcting the foot deformity is ankle arthrodesis, there is no reason that the foot cannot be made stable in a plantigrade position.

Johnson and Haideri [47], using gait analysis, showed that patients in whom lengthening has resulted in plantigrade feet and well-aligned tibiae have better ankle push-off strength and better knee flexion strength than do patients who have undergone Syme’s amputation. These authors noted that the lengthened limb, even if it was stiff and weak, was less different from its opposite normal limb than was the prosthetic side in cases of Syme’s amputation as compared with its opposite normal limb. They reported that the lengthened limb with a plantigrade foot was “clearly more functional than a prosthetic ankle”.

Naudie et al. [39] achieved satisfactory results in only four of ten cases after lengthening. These authors compared their group with an amputation group and concluded that amputation was preferable to lengthening. The reason for the unsatisfactory outcomes was residual or recurrent foot and tibial deformities. Cheng et al. [48], in a small prospective group of four lengthenings, had the same experience, with unsatisfactory results secondary to recurrent tibial and foot deformities. Both groups succeeded in achieving the limb lengthening amounts desired using the Ilizarov apparatus. These results using the Ilizarov apparatus are not much different from those reported by Choi et al. [38], who used the older Wagner method. In the study of Choi et al. [38], all of the cases of higher grades of FH had unsatisfactory results, which were attributed by the authors to rigid uncorrected equino-valgus deformity of the foot. Satisfactory results were achieved in all except one of the patients with mild FH, a patient who had a rigid equino-valgus foot. Choi et al. [38] also concluded that the more severe grades of FH are not candidates for lengthening surgery and would be best served with amputation and prosthetic fitting.

Clearly, although limb length can be successfully corrected in most patients, if the foot deformity is left uncorrected initially or if the foot deformity recurs, the final functional outcome will be unsatisfactory [49, 50]. This conclusion is also valid for the treatment of clubfoot and vertical talus deformities. If one examines the few series in the literature that report good functional results after limb lengthening, the predominant difference is that in the final result not only was the leg length discrepancy addressed successfully but the foot deformities were also addressed successfully.

Miller and Bell [45] reported the outcomes of 12 lengthenings in cases of FH. At the time of final follow-up, all limbs had regained full knee motion and all feet were plantigrade. All but three limbs had regained their preoperative range of ankle motion. None of the ankles had residual instability. Despite these excellent final results, 25 complications occurred in the 12 lengthenings, and the patients required eight secondary procedures to treat and correct complications. Gibbons and Bradish [44] lengthened ten tibiae in cases of FH. In all cases, the desired lengthening was achieved and all patients were able to wear normal shoes without orthoses. A plantigrade position was achieved in all feet without persistent ankle instability. Complications occurred in nine of the ten cases, and all were all resolved either surgically or nonoperatively. Several patients required foot deformity correction with soft tissue or bone procedures.

Perhaps the largest series in the recent literature with the longest follow-up duration is that presented by Catagni and Guerreschi [43]. Using the modified Dal Monte classification [12], these authors reported 32, 37 and 20 cases of grade 1, grade 2 and grade 3 FH, respectively, that were treated with lengthenings, all of which led to completed reconstruction. Of the 32 patients with grade 1 FH, 31 required only one lengthening each and one required a second lengthening. Equal leg lengths with a plantigrade foot were achieved in each of these patients. In the 37 patients with grade 2 FH, five patients required three lengthenings each, nine required two lengthenings each, and 23 required one lengthening each. At the end of the reconstruction 35 of the patients had a plantigrade, functional foot, and the remaining two patients had residual valgus deformity, requiring shoes with orthoses. No patient underwent ankle arthrodesis. Thirty-two of the 37 were ultimately able to participate in recreational sports, and five limited their activities as a result of knee stiffness or instability. In the grade 3 FH group, two patients required six stages of reconstruction (a stage referred to as a lengthening or a deformity correction), four required five stages, six required four stages, three required three stages, four required two stages and one required one stage. Of these 20 patients, eight underwent foot deformity correction as a separate procedure before the age of 3 years. The final result was that 16 feet were plantigrade, stable and asymptomatic, and five had residual valgus with stiffness, requiring an orthosis to alleviate the symptoms. Although most of the patients could bike or swim, athletic pursuits were more limited than in the grade 1 and 2 patients. There were no permanent sequelae of knee subluxation, hip subluxation, nerve injury, nonunion or osteomyelitis in any patient. All of the patients were satisfied with the functional results of their reconstruction.

Paley presented (unpublished results presented at AAOS 1999, Anaheim, California) similar results to those of these last three studies. Excellent functional results, including the desired goal of lengthening, were achieved in 36 of the 38 legs lengthened. The one patient who was rated as having achieved only a fair result had a residual equinus deformity with a painful arthritic ankle and required an ankle fusion. Many patients were involved in recreational and/or competitive athletics. All of the adults in the series were gainfully employed, including the one who required an ankle fusion. Despite complications, the final result was not related to the complication rate. Few of the complications lead to major sequelae; those that do can usually be resolved surgically [51].

One of the other criticisms of lengthening is the psychologic impact on the child. Although lengthening is undisputedly stressful for the child and the family, two recent studies have shown that the majority of problems are transitory and remit with appropriate treatment [52, 53] and that the lengthening treatment does not cause long-term psychologic maladjustment [53]. Although most patients tolerate the lengthening process well, some patients do develop loss of appetite, weight loss and difficulty sleeping. A single small dose of amitriptyline before bedtime is useful in helping these patients. Lengthening should not be an excruciatingly painful experience. If a patient is complaining of a lot of pain, especially during the day while at rest, the cause of the pain should be sought. Pain may be related to pin infection, pin loosening or cutting out, frame instability, nerve entrapment/stretch, reflex sympathetic dystrophy, rupture of the regenerating bone after premature consolidation, among other causes. Appropriate treatment, such as antibiotics, pin removal, wire retensioning, slowing distraction, pin replacement and backing up of the distraction, should be administered as soon as the problem is recognized. Peroneal nerve release should be considered if evidence of peroneal nerve stretch does not respond to slowing distraction.

To minimize the psychologic impact of lengthening, serial lengthenings and surgical reconstructions should be spaced apart according to the patient’s age to allow the child as much time as possible without surgery between sessions. Regarding lengthening, this author’s protocol is to perform the first lengthening when the patient is between 1.5 and 4 years of age, the second lengthening at between 6 and 10 years of age and the final lengthening at between 12 and 14 years of age. Children between the ages of 4.5 and 6 years have the most psychologic difficulty with lengthening, whereas children 4 years and younger have the easiest time with the treatment [21]. This author prefers to complete the last lengthening before the patient is in high school, for social reasons, if possible. Cost is another argument for reconstruction rather than amputation.

In 1988 Johnson and Haderi [47] reported that the cost of amputation and prosthetic fitting from age 1 to 18 years was US $81,000 per patient. In 1994 Williams projected lifetime total costs to be US $373,051 per amputee [54]. During the same time period, the cost of surgical reconstruction was $40,000–50,000 for a single surgical lengthening reconstruction. Thus, even three such reconstructions cost less than the lifetime cost per amputee. Therefore, limb salvage is more cost-effective than amputation. While prices have gone up in the last 20 years since these studies were published, they have likely increased proportionately, and the cost of surgical reconstruction today is likely still less than the lifetime cost of amputation with lifetime prosthetic costs.

Paley et al. compared 22 patients personally treated by the first author with the SUPERankle procedure combined with lengthening to an age-matched group of patients who underwent Syme’s amputation at the Dallas at Texas Scottish Rite Hospital [36]. The results of the comparison demonstrated no difference in function between the two groups. Both groups of patients were satisfied with their results, were equally and functionally active and had no pain. Both groups assessed their function as comparable to normal. The choice is therefore that of the parents as to which procedure they prefer for their child. With lengthening reconstruction surgery using the SUPERankle and lengthening, the big advantage is that in addition to normal function, the patient retains a sensate foot that can feel the ground, thereby providing balance and proprioception. No prosthesis provides sensibility or proprioception. Furthermore, the child and later the adult with the prosthesis must have an expensive high-quality technically advanced prosthesis made every year throughout childhood and frequently every year throughout adult life. This is an important economic consideration. The total cost to health care of these many prosthetic changes is much greater than all of the medical costs related to the surgery of lengthening reconstruction surgery [47, 54]. This does not even factor in the frequent adjustments and modifications to the prosthesis that are required, nor the intermittent skin irritation of the stump to the prosthetic that causes some pain and suffering and sometimes interrupts prosthetic use. It also does not factor in that children and adults with FH with missing knee ligaments who have added stress due to the lever arm of a prosthesis can develop secondary problems at the knee joint. As well, it does not take into account the psychologic effects of having a prosthesis, such as going to the beach and having to take off the prosthetic to get into the water, the impact on dating, or any psychologic stress to the individual with the prosthesis created by not feeling comfortable wearing short pants or skirt. With lengthening reconstruction surgery, these are not considerations that the patient has to deal with.

Patients after SUPERankle procedures and lengthening surgery are able to participate in a wide range of sports, such as baseball, football, basketball, tennis, soccer, gymnastics, rock-climbing, etc. Therefore, the decision to undergo the procedure is a personal one and not one that should be dictated by the surgeon. The option of amputation is too readily provided because of the lack of training and availability of the SHORDT and SUPERankle procedures. While every pediatric orthopedic surgeon has been trained in amputation techniques and while amputation is not a technically difficult procedure, the SHORDT and SUPERankle procedures are technically challenging operations. Since FH is a rare diagnosis (less than 1:50,000 births) and since type 3 FH, which needs to be treated using the SUPERankle procedure, is even more rare, the majority of the pediatric orthopedic surgeons do not see many of these cases. In order to become proficient with the various variations of the SUPERankle procedure, for the different Paley types, one needs to perform this operation several times a year. Most pediatric orthopedic surgeons do not see more than one or two cases of this condition in a year. Therefore, it is difficult, if not impossible, for most pediatric orthopedic surgeons to gain sufficient experience with this procedure even if they do obtain proper training. On the other hand, to be proficient in Symes amputation is far easier since there are many more indications and the procedure is simpler and more forgiving. Therefore, when one takes into account both the lack of training and experience of pediatric orthopedic surgeons and the rarity of the condition, the SUPERankle procedure will remain an obscure, underutilized operation than it should be. It is therefore less likely to be recommended to most patients. Hopefully, with greater awareness centers of excellence can develop this expertise, and it will be offered as an alternative and perhaps one day replace amputation surgery for FH.

Based on this author’s experience, there are few contraindications to lengthening. All patients should be given the option of lengthening reconstruction surgery versus amputation. If the lengthening option is not available at the treating center, patients should be offered a second opinion at a referral center that has expertise in lengthening reconstruction surgery. Socioeconomic factors may limit such second opinion options. Nevertheless, this should be the patient’s decision and not the doctor’s. There are many avenues to overcome socioeconomic limitations in today’s society. In many developing nations, amputation may be culturally unacceptable and good prosthetics unobtainable. In such situations, amputation is contraindicated. Finally, when there are upper extremity deficiencies, which make independently getting in and out of a prosthetic challenging, amputation is also contraindicated [13].

## Conclusion

In conclusion, the final result of lengthening for FH is dependent on the final foot position after reconstruction. It is essential to obtain a plantigrade stable foot to ensure a satisfactory result. The SHORDT or the SUPERankle procedures are the best method to obtain and maintain a plantigrade stable foot. The few cases that are too dysplastic or fail these procedures can be salvaged successfully with ankle fusion. Serial lengthenings and epiphysiodesis, performed at well-spaced intervals during childhood, will equalize the leg length discrepancy. In light of these results all patients should be given the option of surgical reconstruction versus amputation.
